# Wnt/beta-Catenin Signaling and Small Molecule Inhibitors

**DOI:** 10.2174/138161213804581837

**Published:** 2012-02

**Authors:** Andrey Voronkov, Stefan Krauss

**Affiliations:** SFI-CAST Biomedical Innovation Center, Unit for Cell Signaling, Oslo University Hospital, Forskningsparken, Gaustadalleén 21, 0349, Oslo, Norway

**Keywords:** β-catenin, cancer, drug discovery, small molecule inhibitors, stem cells, Wnt.

## Abstract

Wnt/β-catenin signaling is a branch of a functional network that dates back to the first metazoans and it is involved in a broad range of biological systems including stem cells, embryonic development and adult organs. Deregulation of components involved in Wnt/β-catenin signaling has been implicated in a wide spectrum of diseases including a number of cancers and degenerative diseases. The key mediator of Wnt signaling, β-catenin, serves several cellular functions. It functions in a dynamic mode at multiple cellular locations, including the plasma membrane, where β-catenin contributes to the stabilization of intercellular adhesive complexes, the cytoplasm where β-catenin levels are regulated and the nucleus where β-catenin is involved in transcriptional regulation and chromatin interactions. Central effectors of β-catenin levels are a family of cysteine-rich secreted glycoproteins, known as Wnt morphogens. Through the LRP5/6-Frizzled receptor complex, Wnts regulate the location and activity of the destruction complex and consequently intracellular β- catenin levels. However, β-catenin levels and their effects on transcriptional programs are also influenced by multiple other factors including hypoxia, inflammation, hepatocyte growth factor-mediated signaling, and the cell adhesion molecule E-cadherin. The broad implications of Wnt/β-catenin signaling in development, in the adult body and in disease render the pathway a prime target for pharmacological research and development. The intricate regulation of β-catenin at its various locations provides alternative points for therapeutic interventions.

## INTRODUCTION

Wnt/β-catenin signaling is a branch of an extensive functional network that developed around a class of proteins - called armadillo proteins - that dates back to the first anaerobic metazoans. Wnt/β-catenin signaling is involved in a broad range of biological systems, including stem cells biology, developmental biology, and adult organ systems. 

The first detail of the Wnt/β-catenin network was reported in 1982 with the identification of the proto-oncogene int-1 in mice [1]. Later its homolog in *Drosophila*, Wingless, was shown to be required for proper wing formation [2]. In 1989 injection of Wnt1 mRNA in *Xenopus* was shown to cause body axis duplication, and demonstrated the functional conservation of the pathway [3]. Since then, the functional importance of Wnt/β-catenin signaling has been shown in a plethora of developmental and organ systems including the cerebral cortex, the hippocampus, the eye, the lens, the spinal cord, limbs, bone, cartilage, somites, the neural crest, skin, teeth, the gut, the lungs, the heart, the pancreas, the liver, the kidneys, the mammary glands, the hematopoetic system and the reproductive system [4-7]. Deregulation of Wnt/β-catenin signaling is implicated in a wide spectrum of diseases including degenerative diseases, metabolic diseases and cancer [4], [8-11]. 

The key mediator of Wnt signaling, the armadillo protein β-catenin, is found in a dynamic mode at multiple subcellular localizations, including junctions where it contributes to stabilize cell-cell contacts, the cytoplasm where β-catenin levels are tightly controlled by protein stability regulating processes and the nucleus, where β-catenin is involved in transcriptional regulation and chromatin interactions. Central extracellular regulators of β-catenin levels are the Wnt morphogens. However, multiple other processes, including hepatocyte growth factor, prostaglandines, PKA (Protein Kinase A), E-cadherin, and hypoxia, can also influence β-catenin levels.

β-catenin itself is a specialized member of the larger armadillo protein family that consists of three subfamilies: the p120 subfamily, the beta subfamily (β-catenin and plakoglobin) and the more distant alpha subfamily. The functional interplay between members of this protein family is not well understood, but an involvement of p120 and plakoglobin in Wnt/β-catenin signaling has been shown.

The regulation of the presence and stability of β-catenin and functionally convergent armadillo proteins – in particular p120 - at the various cellular localizations as well as their shuffling within the cell provides alternative intervention points for therapeutic reagents. The broad implications of Wnt/β-catenin signaling in development, the adult body and in disease renders it a prime target for pharmacological research and development. A short overview map for canonical Wnt signaling is presented on Fig. (**1**).

The armadillo protein β-catenin is the central denominator of Wnt/β-catenin (canonical Wnt) signaling. The levels of β-catenin at different subcellular localizations are regulated by a variety of processes including site-specific phosphorylation of β-catenin. In particular, the control of the turnover of cytoplasmic β-catenin by the destruction complex and the control of the destruction complex by the Wnt signalosome have been studied extensively. Other important mechanisms regulating subcellular β-catenin thresholds are those controlling its mobilization from adherens junctions and its translocation to the nucleus. One of the central end points of the Wnt/β-catenin signaling pathway is the regulation of transcription through the binding of β-catenin to members of the Tcf-1/lymphoid enhancer factors (Lef-1, 3, 4) family of transcription factors in the nucleus [12-14]. 

The structure of β-catenin can be divided into three domains: the N-terminal domain, the armadillo domain consisting of 12 armadillo repeats, and the C-terminal domain [15]. Through predominantly positively charged armadillo (Arm) repeats, β-catenin is a member of an expanded and evolutionary ancient protein family that includes plakoglobin (γ-catenin), APC (adenomatosis polyposis coli), p120 and other proteins [16-18]. Local charge alterations of β-catenin through phosphorylation at a multitude of positions have been suggested to regulate its affinity to specific protein partners. This includes C-terminal phosphorylation that attenuates the binding of β-catenin to the cadherin adhesion complex and N-terminal phosphorylation that regulates its degradation in the proteasome. Furthermore, phosphorylation regulates the association of β-catenin with Tcf/Lef during transcriptional regulation [19]. 

In this review we will first describe the alterations of β-catenin in the destruction complex and the proteins that are involved in this process. We will then focus on the Wnt signalosome that recruits components of the destruction complex thus inactivating it. Subsequently, we will summarize how the signalosome is removed from the cell surface by endocytosis. Next we will describe the cellular pool of β-catenin at cell junctions and the mobilization of β-catenin from this pool. Then we will focus on the implications of β-catenin in transcription control. Finally, we will summarize some of the pathways that influence Wnt/β-catenin signaling. Several proteins in the Wnt/β-catenin pathway that are implicated in other cellular processes will be briefly described.

### The β-Catenin Destruction Complex and its Proteins

In the absence of an active Wnt signalosome, cytoplasmic β-catenin associates with the destruction complex. The main known structural components of the destruction complex are APC and Axin. To this structural core, the casein kinases CK1α, δ, and ε (to be referred to collectively as CK1) and GSK3 (glycogen synthase kinase 3) are recruited [20].

In the degradation complex the processing of β-catenin is considered as a phosphorylation-dependent flux along the Axin scaffold protein which is regulated by a stepwise series of phosphorylations triggered by the kinases CK1 and GSK3 [15, 20]. A current model proposes that β-catenin initially binds to Axin. The priming kinases CK1 phosphorylate β-catenin at Ser45 [20], which enables a subsequent phosphorylation by GSK3 at Ser33, Ser37 and Ser41 [21, 22]. Subsequent phosphorylation of APC by CK1ε and GSK3, leads to an increased affinity between APC and β-catenin [23] triggering a transfer of β-catenin from Axin to APC, while Axin is able to bind the next β-catenin molecule. Finally, APC exposes the N-terminally phosphorylated β-catenin to β-TrCP (b-transducin-repeat-containing protein) [24], the ubiquitin ligase responsible for ubiquitinylating β-catenin leading to its degradation in the proteasome [25]. N-terminal phosphorylation of β-catenin is not only required for its degradation, but also responsible for attenuating its effect on transcription [20, 26, 27].

### Axin

In diverse human cancers Axin mutations are associated with increased levels of β-catenin [28-30]. Mutations in the genes encoding Axin and Axin2 are found in 11% of cases of colorectal cancers and in hepatocellular carcinomas [31-33]. Furthermore, mutations in the Axin gene are observed in 12% of cases of medulloblastomas, in 35% of cases of adenoid cystic carcinomas and in 20% of cases of oral squamous cell carcinomas [34].

In the destruction complex, Axin serves as a coordinating scaffold for the kinases GSK3 and CK1, for the structural protein APC and Dishevelled (Dvl/Dsh) as well as for β-catenin [35]. Early mathematical kinetic modeling for Wnt/β-catenin signaling suggested that Axin levels may be the rate-limiting factor for the degradation of β-catenin. These models were based on the assumption that Axin concentrations are about three orders of magnitude lower than the concentration of other degradation complex components known at the time of the study [36]. Since Axin is considered to be a rate limiting protein in the destruction complex, strategies involving an alteration of Axin protein levels are considered to be promising in drug discovery [36-40]. 

There are two Axin proteins in humans - Axin1 (826aa, 92kDa predicted in humans), and Axin2 (also called conductin or Axil, 840aa, 93kDa). Each of the Axin genes encodes two isoforms, a and b, which differ by splicing variants [34]. Axin and Axin2 have redundant functions in Wnt/β-catenin signaling, both binding to various proteins of the β-catenin degradation complex [41, 42]. The transcription of Axin2 is a central target of Wnt/β-catenin signaling, whereby Axin2 forms a negative feedback loop in the pathway [43]. Hence, Axin2 expression is upregulated by Wnt/β-catenin signaling while Axin contributes centrally to the degradation of β-catenin [44]. 

Axin contains a number of domains including a RGS (Regulators of G protein signaling) domain (aa 121 to 247) and a DIX domain (Dishevelled/Axin homologous domain) (aa 716 - 900). The DIX domain is responsible for Axin homodimerization and the formation of heterodimers with Dishevelled [45, 46]. In this process, the residues 757-820 of the Axin DIX domain bind to the homologous DIX domain of Dishevelled [46-48]. In addition to heterodimerization through the DIX domain, Axin was shown to have two further domains – D and I - that can mediate homodimerization [49]. The RGS domain of Axin interferes with the α-subunits of G-proteins (guanine nucleotide-binding proteins) [50, 51]. Gα proteins were reported to disrupt interactions between Axin and GSK3 [52, 53] and in *Drosophila* it was shown that the α-subunit of G_o_ physically binds to Axin and recruits it to the plasma membrane [54]. Axin uses aa 437-506 to interact directly with the Armadillo repeats 2-3 of β-catenin [55]. The same repeats were also shown to interact with the armadillo protein plakoglobin [56]. Other binding areas in the Axin protein are amino acids 89–216 for APC, 507-712 for Axam, 353–437 for GSK3, 530–712/757–820 for Dishevelled, 217-352/508-712 for CK1 and 353-437 for Diversin [35]. Recently it was also shown by X-ray analysis that the N-terminal domain of Axin (1-80 aa) is responsible for binding to Tankyrase [57]. Interactions with Axin promote dimerizatoin of Tankyrase. Additional Axin-interacting proteins include MEKK1 (MAP kinase kinase kinase), MEKK4 (MAP kinase kinase kinase 4), CK1α/ε, I-mfa (inhibitor of myogenic basic helix-loop-helix transcription factors), Axam (Axin associating protein), PP2A (Protein phosphatase 2), Smad3 (Mothers against devapentaplegic 3), LRP5/6 (Low-density lipoprotein receptor-related proteins 5/6), MEKK4, Ccd1(Coiled-coil-DIX1) and PIAS (Protein inhibitor of activated STAT) [35].

Axin thresholds and stability are regulated by different components of the Wnt/β-catenin pathway. Axin is stabilized by GSK3-mediated phosphorylation at Ser330, Thr341 and Ser343 [35, 58]. In mice, GSK3-mediated phosphorylation of amino acids Thr609 and Ser614 of Axin has been shown to be required for its activity [59]. Axin phosphorylation by GSK3 and CK1 also leads to increased affinity for β-catenin and enhances the phosphorylation and degradation of β-catenin [59-61]. Since the armadillo domain of β-catenin is positively charged in the area that mediates Axin interactions, phosphorylation of Axin can enhance the interaction, while a subsequent N-terminal phosphorylation of β-catenin adds a negative charge that presumably triggers its dissociation from the phosphorylated Axin [20]. 

Axin can be dephosphorylated by the serine/threonine phosphatases PP1 (Protein Phosphatase 1) and PP2C (Protein Phosphatase 2C) [60, 62] and the Ser/Thr phosphatase PP2A, which binds to aa 508-712 and aa 298-506 of Axin [35, 45, 55, 63-65]. PP2A was also reported to dephosphorylate APC [66]. PP1 acts on Ser residues of Axin to reverse CK1α-mediated phosphorylation. Hence, inhibition of PP1 can lead to an increased phosphorylation of Axin followed by an enhancement of β-catenin degradation [60]. Phosphatases therefore may be a targetable interferrence point of Wnt/β-catenin signaling. For instance the phosphatase inhibitor okadaic acid (Table **1**) reverses LiCl (inhibitor of GSK3) induced activation of Wnt/β-catenin signaling [58], and the PP1 inhibitor tautomycin (Table **1**) was shown to reduce Wnt/β-catenin signaling [60].

Axin protein stability and turnover in the cell is centrally regulated by poly(ADP-ribosyl)ation, followed by ubiquitination and protein degradation in the proteasome. Axin ubiquitination is induced by the E3 ubiquitin ligases RNF146 (RING finger protein 146) that recognizes poly(ADP-ribosyl)ate tails at the protein that are added dynamically by the PARP (Poly (ADP-ribose) polymerase) proteins Tankyrase1 and Tankyrase2 [67, 68]. In contrast, SUMOylation was shown to prevent Axin polyubiquitination and thus to stabilize Axin. SUMOylation of Axin occurs at residues K951 and K954 in the C-terminal KVEKVD sequence and is implemented by E3 ligases of the PIAS family [69]. SUMOylation does not only regulate Axin stability, but also its subcellular localization [69]. Axam downregulates Wnt/β-catenin signaling [70] by binding to residues 507-712 of Axin and deSUMOylates the protein [35, 69-71]. Interestingly, Axam is also involved in deSUMOylation of Tcf-4 [72]. Another Axin2-interacting protein that has shown to regulate the stability of Axin, is the arginine methyltransferase PRMT1. PRMT1 directly interacts with Axin and methylates Arg378, resulting in a stability increase of Axin and leading to a reduction of Wnt/β-catenin signaling [73].

Three nuclear localization signal sequences (NLS) are found in the Axin proteins at positions 443-558, 474-483 and 537-547. Because Axin lacking NLS fails to regulate cytoplasmic levels of β-catenin it has been suggested that Axin may serve as a shuttle for β-catenin between the cytoplasm and the nucleus [74]. Interestingly, it has also been shown that Axin may act as a molecular shuttle to export β-catenin from the nucleus [74], and that this function may require Axin oligomerization into larger aggregates [74]. Finally, Axin as well as several other components of the degradation complex (GSK3, β-catenin, Tankyrase and APC) may co-localize to centrosomes and mitotic spindles [75-78], where Axin modulates the distribution of Axin associated-proteins such as PLK1 (Serine/threonine-protein kinase, also known as polo-like kinase 1) and GSK3, thereby modulating the mitotic process [79].

The structural protein Axin participates not only in Wnt/β-catenin signaling but also in TGFβ (Transforming growth factor beta**) **signaling and MAPK (Mitogen-activated protein-kinase)-mediated signaling [35]. An overexpression of Axin has been reported to lead to an activation of MAP kinase (Mitogen-activated protein kinases) and the c-Jun N-terminal kinase JNK. In TGFβ signaling, Axin assists in TGFβ-mediated activation of Smad3 [80]. Smad3 in turn can activate β-catenin signaling through a direct interaction with β-catenin whereby Smad3 protects β-catenin from ubiquitination and degradation [81].

### APC

APC is the largest structural core protein of the destruction complex (2843 amino acids, 312 kDa). The APC protein has several functional domains including an oligomerization domain (responsible for homodimerization), seven armadillo repeats and three β-catenin binding repeats of 15 amino acids [82, 83]. The β-catenin binding repeats were proposed to bind β-catenin and assist in its positioning to the binding sites of the kinases in the destruction complex. In addition, APC has seven 20 aa repeats that are involved in release of β-catenin after its phosphorylation [15, 24]. In most cases, oncogenic mutations in the gene encoding APC are caused by a truncation of the β-catenin binding region [84, 85]. However, APC mutations that do not affect β-catenin binding may also be cancerogenic e.g. if they lead to a reduction of Axin/APC binding and thus to destabilization of the destruction complex [9, 86]. 

APC can be phosphorylated by CK1ε at Ser1279 and Ser1392 [87]. Phosphorylated APC outcompetes Axin from forming a complex with β-catenin and it has been suggested that the synchronized coordination between Axin, β-catenin and APC phosphorylation is important for a stepwise processing of β-catenin in the degradation complex [20, 88].

Similar to Axin, APC was found to act as a nuclear shuttling protein and has been implied in nuclear β-catenin import as well as export [89, 90, 91]. APC has two nuclear localization signals (NLS), which use the importin α/β-system to shuttle APC into the nucleus [92]. It was shown that phosphorylation of APC at Ser2054 (C-terminal of the second NLS) negatively regulates APC transport to the nucleus [92]. Curiously, APC which lacks the NLS can still enter the nucleus [93] and it was reported that B56α, the catalytic subunit of PP2A, facilitates the nuclear transport of APC [93]. Nuclear APC was found to negatively regulate β-catenin-mediated transcription [94]. 

Among other cytoplasmic proteins that interact with APC are plakoglobin (γ-catenin) [95], tubulin [96], EB1 (microtubule-associated protein of the RP/EB family) [97] and hDLG (human disks large homolog 1) [94, 98]. In the nucleus APC has been shown to interact with DNA polymerase β, proliferating cell nuclear antigen (PCNA), the protein tyrosine phosphatase (PTP-BL) [94], the transcription factor activator protein AP-2alpha and the nuclear export factor Xpo1 (Exportin 1) [94].

### GSK3 

Glycogen synthase kinase-3 (GSK3) was initially identified as a serine/threonine protein kinase, which phosphorylates glycogen synthase in rabbit skeletal muscles leading to an inhibition of glycogen synthesis [99]. In humans there are two isoforms, GSK3α (483 aa, 51kDa) and GSK3β (433 aa, 47kDa), that are encoded by different genes. The two isoforms have high amino acid sequence identity (97%) in the catalytical domain, but are less conserved otherwise. GSK3β has two splicing isoforms, one containing a 13 aa insertion (GSK3β2) [100]. Although mutations in GSK3 are usually not associated with cancers, downregulation of GSK3 has been observed in hepatocellular carcinoma, squamous cell carcinoma and prostate cancer [101-103]. However, GSK3 was also suggested as anti-cancer biotarget [104]. GSK3 is involved in a large number of cellular processes [104-107]. A knockout of GSK3β in mice leads to embryonic lethality and is not compensated by GSK3α [108]. Although GSK3 recognition sequences can be found in almost half of all human proteins, a recent overview provides a list of 77 validated substrates of GSK3 [109]. These substrates can be clustered into several functional subsets: inflammation, cellular proliferation, structural rearrangements and glucose metabolism. Importantly, GSK3 appears to be involved in decision points between maintaining stem cell properties, and triggering differentiation. Inhibition of GSK3 together with inhibiting FGF-MAPK (FGF - Fibroblast Growth Factor) signaling enables long-term propagation of embryonal stem cells in mice [110]. Furthermore, deletion of both GSK3α and GSK3β in the brain increases self-renewal of neuronal progenitor cells, while neurogenesis is downregulated [111].

In the context of Wnt/β-catenin signaling, the GSK3α and GSK3β isoforms were shown to be fully redundant [112] and thus will be referred to herein collectively as GSK3. However, in other cellular processes GSK3α and GSK3β may not fully compensate each others functions [113, 114].

An involvement of GSK3 in Wnt/β-catenin signaling was first shown in *Xenopus laevis* embryos, where a mutated GSK3β induced a ventral axis duplication indicative of overactive canonical Wnt signaling [21, 115]. In contrast, active GSK3 was shown to negatively regulate Wnt/β-catenin signaling through an N-terminal phosphorylation of β-catenin in the destruction complex [116-118]. Interestingly, plakoglobin can also undergo GSK3-dependent phosphorylation and proteasomal degradation [119-120]. In the case of β-catenin, GSK3 requires a priming kinase that acts on a 4-5 amino acid C-terminal to a GSK3 phosphorylation site. Phosphorylated amino acids of the priming site bind to the catalytic pocket in GSK3β, formed by the amino acids Arg96, Arg180 and Lys205 and facilitate further phosphorylation through GSK3 [121]. 

The kinase activity of GSK3 can be attenuated by a phosphorylation of Ser21/Ser9 (GSK3α/GSK3β) through different kinases: protein kinase A (PKA), Akt/PKB (protein kinase B), PKC (protein Kinase C), p90 ribosomal S6 kinase/MAPK-activating protein (p90RSK/MAPKAP) and p70 ribosomal S6 kinase (p70S6K) [107]. p38 MAPK (p38 mitogen-activated protein kinases) can selectively reduce the kinase activity of GSK3β, but not GSK3α through a phosphorylation of Thr390, which can lead to reduced β-catenin degradation [122]. In contrast, autophosphorylation of GSK3α/ GSK3β at Tyr279 or Tyr216 respectively can enhance the activity of GSK3 [107, 123].

There are multiple further protein/protein interactions that can modulate GSK3 activity. FRAT1 (frequently rearranged in advanced T-cell) and FRAT2, members of the GSK-3-binding protein family, compete with Axin for GSK3 binding and hence inhibit the activity of GSK3 in the context of Axin [124-126] whereby the Axin binding site on the GSK3 protein overlaps with the binding site for FRAT. Also, Dishevelled can interact with FRAT1, recruiting it to a ternary complex between Dvl, Axin and GSK3. This complex leads to an inhibition of GSK3 and consequently to a stabilization of β-catenin and an activation of Wnt/β-catenin signaling [125]. FRAT1 overexpression is associated with tumorigenesis [127-130]. Interestingly, FRAT1 is considered to be one of the links between β-catenin dependent (canonical) and β-catenin independent (non-canonical) Wnt signaling through its activation of JNK and AP-1(activator protein 1) [131].

In addition to a direct involvement in regulating β-catenin stability by phosphorylation, GSK3 has also a pleothora of indirect implications on Wnt/β-catenin signaling, predominantly synergizing with its function in antagonizing Wnt/β-catenin signaling. In particular GSK3 has both an effect on the transcriptional regulation of β-catenin, and on central β-catenin target genes. The oncogene c-Myc is among the primary target genes that are upregulated by β-catenin/Lef. GSK3 phosphorylates the Thr58 residue of c-Myc leading to a reduction of its half-life [109]. Importantly, GSK3 phosphorylates Ser resides in the oxygen-dependent degradation domain of the transcription factor Hypoxia-inducible factor 1α (HIF-1*α*)**that links hypoxia to β-catenin-mediated signaling. An inhibition of GSK3 promotes HIF-1*α* stability while an upregulation of GSK3 has an opposite effect [132]. Another interesting substrate of GSK3 with implications on β-catenin-mediated signaling is the zinc-finger transcription factor Snail, which represses the transcription of E-cadherin. An inhibition of GSK3 leads to an upregulation of Snail followed by a down-regulation of E-cadherin which could lead to a cytoplasmic mobilization of β-catenin [133]. A more detailed description of GSK3 and GSK3 inhibitors is given in [104].

Numerous small molecular GSK3 inhibitors have been reported [134, 135]. Most GSK3 inhibitors target the ATP-binding site in the catalytic domain of the protein, which has 86% amino acid identity to the ATP-binding sites of CDK1 (cyclin-dependent kinase 1) and other kinases [107]. Hence, most of the published GSK3 inhibitors show low selectivity for GSK3. However, inhibitors that target the substrate binding site of GSK3 with increased specificity, are also reported [136].

There are also possibilities for increasing the activity of GSK3 by pharmacological intervention. Phosphorylation of GSK3 by p38 MAPK on Thr390 reduces the activity of the GSK3 kinase. Accordingly, small molecular inhibitors of p38 MAPK (SB203580 or SB239063, Table **1**) can lead to increased GSK3 activity and in consequence reduced Wnt/β-catenin signaling [122, 137]. Interestingly, both compounds affect only GSK3β, but not GSK3α, making the intervention isoform-specific. Several p38 MAPK inhibitors are in clinical trials including the anti-inflammatory drug PH-797804 and dilmapimod [138, 139].

### Tankyrases

There are two Poly (ADP-ribose) polymerases (PARPs) that are implicated in Wnt/β-catenin signaling: Tankyrase 1 (PARP5a) and Tankyrase 2 (PARP5b) [140, 141]. To a large extent, Tankyrases 1 and 2 appear to have redundant functions.

Tankyrase 1 has four functional domains: the HPS domain (consisting of His, Pro and Ser repeats), Ankyrin domain (consists of 20 ankyrin repeats), a SAM (sterile alpha motif) domain and the catalytic PARP domain. In contrast to Tankyrase 1, Tankyrase 2 lacks the HPS domain [141]. The PARP domain catalyzes poly(ADP-ribosyl)ation, the SAM and Ankyrin domains participate in the formation of protein-protein complexes with substrates, while the functions of the HPS remain obscure. One of the central properties of Tankyrases is their capability to form dynamic oligomers predominantly through their SAM domain, but presumably also assisted by the ankyrin domains, and to subsequently destabilize such oligomers through increasing, context depending poly(ADP-ribosyl)ation [142-146]. The ability of Tankyrase to form dynamic multimers has led to the suggestion that Tankyrase oligomers can regulate the assembly and disassembly of large polymerized structures in response to signals [145]. In the context of the destruction complex, poly(ADP-ribosyl)ation of Tankyrases, and possibly Axin appear to trigger deoligomerization due to an accumulation of negative charges and repulsive forces [145]. Through poly(ADP-ribosyl)ation Tankyrases also regulate a number of further protein complexes, including complexes involving IRAP (insulin-responsive amino peptidase), NuMa (nuclear mitotic apparatus protein 1), Mcl-1 (myeloid cell leukaemia 1), EBNA-1 (Epstein-Barr nuclear antigen1), TRF1 (telomeric repeat binding factor 1), TAB182 (Tankyrase 1 binding protein 182) and GRB14 (growth factor receptor-bound protein 14). A recent overview of Tankyrase substrates is provided in [146].

Poly(ADP-ribosyl)ation is a catalytic reaction whereby nicotinamide adenine dinucleotide (NAD^+^) is used as a substrate to create multimeric side chains. At the first step, in the catalytic PARP domain, the nicotinamide part of NAD+ interacts with the Gly1032 (human Tankyrase 2 amino acid sequence numbering) residue of Tankyrase. At the second step, ADP-ribose is transferred from NAD^+^ to a glutamic acid residue of the target protein. Next, a further monomer is added to the polymer via the same mechanism using the hydroxyl group of the previous monomer. Poly(ADP-ribosyl)ation may include branching points in a process that is hiterto poorly understood. In the context of the destruction complex, poly(ADP-ribosyl)ated proteins appear to interact with the ubiquitin ligase RNF146 [37, 67, 68]. 

Poly(ADP-ribosyl)ation is a reversible process since poly(ADP-ribose) polymers may be removed by poly(ADP-ribose) glycohydrolase (PARG). It was recently suggested that in this process the Glu115 residue of PARG (*T.curvata*) replaces the ribose moiety from an ester followed by a replacement of Glu115 by a water molecule [147]. A PARG inhibitor, ADP-HPD was shown to decrease Tankyrase stability [148, 149]. 

The involvement of Tankyrase in attenuating the destruction complex was described [37]. In the process, Tankyrase poly(ADP-ribosyl)ates Axin. Poly(ADP-ribosyl)ated Axin is then recognized by the RNF146 ubiquitin ligase followed by ubiquitination and degradation [37, 67, 68]. The interaction of RNF146 with the poly(ADP-ribose) tail of Axin appears to be mediated through a recognition of the iso-ADP-ribose moiety (but not ADP-ribose) by the WWE domain of RNF146 [150]. In parallel, Tankyrase auto poly(ADP-ribosyl)ation also leads to RNF146-mediated ubiquitination and subsequent degradation [67, 68]. Furthermore, RNF146 is poly(ADP-ribosyl)ated and ubiquitinated [68]. The HECT-type ubiquitin E3 ligase HUWE1 associates to RNF146 and was suggested to participate in ubiquitin chain elongation [68]. A number of RNF146-interacting proteins were identified, including PARP1, PARP2 and three proteins involved in DNA-damage response [68]. Noteworthy, RNF146 prevents Tankyrase co-localization to centrosomes [68].

To date it remains unclear whether Tankyrase presence and stability in the degradation complex can be regulated by mechanisms other than poly(ADP-ribosyl)ation, and whether such regulation might be dependent on components of Wnt/β-catenin signaling. Several kinases are known to be involved in Tankyrase phosphorylation: GSK3, PLK1 and MAPK. PLK1 complexes with Tankyrase1 both *in vivo* and *in vitro* and activates Tankyrase through phosphorylation [151]. Disruption of PLK1 decreases the stability of Tankryase 1 and leads to a reduction of its PARP activity. Interestingly, phosphorylation of Tankyrase by PLK1 was also shown to affect mitotic spindle assembly (see below) and the regulation of telomeric ends [79]. PLK1 also mediates phosphorylation of Dishevelled2 [152]. MAPK has been shown to enhance the catalytic activity of Tankyrase in the context of IRAP4 [153].

Tankyrase has further cellular functions. It has been shown that Tankyrase is involved in glucose transport. In this process, Tankyrase associates with GLUT4 (glucose transporter type 4) vesicles through binding to the insulin responsive aminopeptidase (IRAP) [153]. The IRAP is required for the targeting of vesicles carrying the glucose transporter GLUT4 [154]. GLUT4 mediates the insulin-stimulated glucose uptake in adipocytes and muscle cells. In this context, Tankyrase acts as a positive regulator of insulin-mediated GLUT4 translocation from cytosolic vesicles to the cell surface to mediate glucose uptake [141]. 

Another role of Tankyrase is its influence on the cell cycle through its interaction with the nuclear mitotic apparatus protein (NuMA), associated to spindle poles in mitosis from prophase to anaphase [155, 156]. NuMa is thought to be an important structural protein both for the nucleus and spindle poles [156]. A Tankyrase knockdown leads to defects in mitotic spindle functions and to defects in the microtubules [141]. GSK3 is involved in mitotic phosphorylation of Tankyrase [157] on Ser978, Thr982, Ser987 and Ser991 in the conserved [S/T]-X-X-X-[S/T] motif. Whether Tankyrase phosphorylation by GSK3 impacts Tankyrase function at the mitotic spindle through NuMa poly(ADP-ribosyl)ation remains to be studied. 

Tankyrases are involved in telomere maintenance by poly(ADP)ribosylating TRF1 (which prevents telomerase activity on telomeres) and releasing TRF1 from telomeres [140, 151]. In this context it is noteworthy that there are further links between telomeres and Wnt/β-catenin signaling. One of them is TERT (telomerase reverse transcriptase), a catalytic subunit of telomerase, that was shown to directly regulate Wnt/β-catenin signal by participating as a co-factor in the β-catenin/Tcf transcriptional complex [158]. It has been shown that an overexpression of either TERT or β-catenin in mouse hair folicles results in a similar phenotype [159, 160, 161]. Hence, under certain conditions gene regulation by TERT and β-catenin might intersect [162].

As many other components of the β-catenin degradation complex, Tankyrase can be observed in the vicinity of the plasma membrane. Such localization is triggered by E-cadherin-mediated cell-cell adhesion, as shown on polarized epithelial MDCK cells [157]. Tankyrase recruitment to the lateral membrane follows a calcium initiated cell-cell adhesion and is reversed by calcium depletion. Inhibition of the poly(ADP)ribosylation of Tankyrase leads to its stabilization and accumulation near the lateral membrane [157]. An inhibition of Tankyrase also leads to an inhibition of EMT *ex *vivo [163], which indicates that Tankyrase may influence intercellular adhesion. Accordingly, the disruption of intercellular adhesion by calcium depletion leads to a Tankyrase release into cytoplasm.

Tankyrases have been identified as a promising target for inhibiting Wnt/β-catenin signaling. Several research groups have identified small molecules that inhibit Tankyrases and correspondingly Wnt/β-catenin signaling (Table **1**) by stabilizing the destruction complex [37-40], [165, 166]. 

Tankyrase inhibitors can be classified into two groups that bind differentially to the PARP catalytic center: one group binds to the nicotinamide pocket whereas the other occupies predominantly the adjacent ADP pocket. The first group includes the Tankyrase selective XAV939, and many generic PARP inhibitors (PDB structures in Protein Data Bank, www.rcsb.org: 3KR8, 3MHJ, 3P0P, 3P0Q, 3MHK and 3U9H) [37, 165, 166]. These compounds usually have stacking interactions with the side chain of Tyr1071 and form two hydrogen bonds with Gly1032 (numbering for human Tankyrase 2). Tankyrase inhibitors that bind to the ADP pocket include IWR1, JW55, and JW74 (Table **1**) [38-40]. These molecules participate in stacking interactions with the side chain of histidine (aa 1201 in Tankyrase 1, aa 1048 in Tankyrase 2) and in hydrogen bonding with the backbone amides of Tyr1213 (Tyr1060 in Tankyrase 2) and Asp1198 (Asp1045 in Tankyrase 2) in the adenine dinucleotide pocket (PDB structures in Protein Data Bank, www.rcsb.org: 1UDD, 1UA9 and 4DVI) [167-169]. An interesting binding mechanism is exerted by the compound PJ34 (PDB code, www.rcsb.org: 3UH2) in that two molecules of PJ34 (Table **1**) can simultaneously bind to the Tankyrase PARP domain; one in the nicotineamide pocket, the other in the ADP pocket [170]. A profound review on ADP-(ribosyl)ation as old and new targets for cancer therapy is given in [171].

### The Wnt receptor complex

The Wnt signalosome is the the best studied system that counteracts β-catenin degradation and enhances β-catenin-mediated signaling. The Wnt signalosome does so by recruiting components of the destruction complex to the membrane, a process that is triggered by binding of one of several Wnt morphogens to the transmembrane proteins Frizzled and LRP5/6. In the process, the Wnt signalosome itself is cleared from the plasma membrane by endocytosis.

Before Wnt morphogens can induce the Wnt signalosome, they mature by undergoing a number of post-translational modifications prior to being secreted. During post-translational maturation, Wnt morphogens undergo N-glycosylation in the endoplasmic reticulum (ER) [172-174], S-palmitoylation of the N-terminal residue Cys77 (mouse Wnt3a) [175] and acetylation with palmitoleic acid at Ser209, which is required for secretion [176, 177]. The functional implications of these post-translational modifications are not entirely understood. For example, some studies suggest that glycosylation is important for secretion, while other studies do not confirm such a link [172], [178]. Although palmitoleic modification may not be strictly required for secretion, it participates in Wnt binding to Frizzled receptors and in Wnt signal transduction [179]. Wnt proteins with a mutation in the cystein that is the target for palmitoylation are not able to transduce Wnt signaling. It has been proposed that the hydrophobicity of palmitate and palmitoleic acid is required for Wnt to interact with cellular membranes, which is necessary for the interaction with Frizzled/LRP5/6 receptors [180]. The lipid modifications as well as the acceptor amino acids are highly conserved among different Wnt proteins in diverse organisms. 

After posttranslational modifications in the ER, Wnt proteins are transported to the Golgi apparatus. From the Golgi apparatus, Wnt proteins are translocated to the cellular membrane with the assistance of the seven-pass transmembrane orphan G-protein coupled receptor Evenness interrupted (Evi)/Wntless(Wls) (GPR177 in mammals) that co-localizes to the Golgi apparatus, cellular membrane and endocytic vesicles [176, 181-183]. Evi/Wntless exports all Wnt proteins [184]. In *Drosophila* it has been shown that acylation of Wnts is required for their binding to Evi/Wls, while glycosylation and S-palmitoylation do not appear to be required [172, 181, 185]. In mammalian cells N-linked glycosylation is required for GPR177 localization to the Golgi apparatus and targeting to the plasma membrane [186, 187]. In mouse, deletion of GPR177 leads to axis formation defects and early fetal lethality [188]. Finally, Evi/Wls is cleared from the plasma membrane by endocytosis in a process that involves the GTPase Rab5 [164]. Clathrin-mediated endoytosis of Evi/Wls and endosomal sorting through the trans-Golgi network (TGN) appear to be required for the proper secretion of Wnt morphogens. Thus, disruption of these processes leads to an Evi/Wls accumulation on the plasma membrane and a downregulation of Wnt secretion [176, 189]. Lipidation and acidification of secretory vesicles was suggested to be important for Wnt secretion [176, 190] and a blockage of v-ATPase-mediated acidification of secretion vesicles leads to an accumulation of the Evi/Wls complex in vicinity of the cellular membrane and downregulates Wnt secretion [185]. Interestingly, the transcription of the mammalian GPR177 gene is enhanced by Wnt/β-catenin signaling [186]. 

Also central to the secretion of mature Wnt morphogens is the multipass membrane protein Porcupine (Porc) that interacts with the N-terminal domain of Wnt [191]. Loss of Porc leads to an accumulation of Wnts in the ER [192]. Porc function is antagonized by the protein Oto, which is a homolog of the *Drosophila* glycosylphosphatidylinositol (GPI)-inositol-deacylase PGAP1. Oto deacetylates Wnt proteins in the secretory pathway, leading to its retention in the endoplasmic reticulum [193]. A class of potent small molecule inhibitors called IWP (Table **1**) that target Porc and thereby inhibit Wnt secretion was identified using high-throughput screening [38]. Diverse IWP analogs were reported recently [194].

Signaling of Wnts is limited to approximately 20 cellular layers from the source of secretion [195]. It has been shown that heparane sulfate proteoglycans (HSPG) are involved in Wnt signaling and stabilizing the activity of purified Wnt proteins through preventing their aggregation [196]. Since Wnt proteins/morphogens are insoluble and highly lipophilic, they require a specialized transport system. One way to transport Wnt proteins are lipoprotein particles, which associate with lipid modified Wnts [197, 198]. Another interesting way to transport Wnts is a direct translocation from cell to cell through a series of exocytosis-endocytosis cycles [199]. Hence, Wnts can be transported between the cells via exosomal vesicles [198, 200]. 

There are several models that describe the binding of Wnt morphogens to the Fz-LRP5/6 receptors to initiate Wnt/β-catenin signaling [7]. Historically, Wnt morphogens and Frizzled receptors were classified as canonical (β-catenin dependent) and non-canonical (β-catenin independent) proteins. However, closer scrutiny revealed that at least some of the Frizzled receptors and Wnt proteins can participate in both β-catenin dependent and independent signaling in a context-dependent manner [201-204]. LRP5 and LRP6 are thought to play redundant roles in the Wnt signalosome and are usually referred to as LRP5/6 [205, 206]. In humans, 19 Wnt morphogens and 10 Frizzled receptors are known to date. Mutations in Frizzled receptors were first identified in mutant *Drosophila* [207]. Later, it was found that Frizzled proteins belong to the family of seven-pass transmembrane receptors and bind Wnts [208, 209]. The most popular model proposes that Wnt binds to the transmembrane protein Frizzled and provides a link to the transmembrane protein LRP5/6. This binding forms the core of the Wnt signalosome and triggers a receptor oligomerization. Hetero-oligomerization of Fz-LRP5/6 is sufficient for activating Wnt/β-catenin signaling as demonstrated elegantly by studies involving chimeric Fz-LRP5/6 and Fz-Dkk proteins [210-212]. Evidence suggests that LRP6, in addition to participating positively in Wnt/β-catenin signaling, may also be engaged in an inhibitory role in β-catenin independent Wnt signaling [213, 214]. 

The oligomerization of the Wnt signalosome is enhanced on the intracellular side of the complex by Dishevelled, which oligomerizes through its DIX domain [215-217]. The Ser/Thr-rich motifs on LRP6 together with Disheveled (Dvl) are then responsible for recruiting Axin and GSK3 to the Wnt signalosome [218-222], inducing Axin polymerization at the cytoplasmic side of the receptor complex [46]. In this process, the lipid kinases PI4KII and PIP5KI have been implicated in the formation of the Wnt signalosome and the translocation of Axin/GSK3 from the destruction complex to the plasma membrane [223]. It was shown in *Drosophila* that the recruitement of Dishevelled and Axin to the membrane is facilitated through G-proteins with trimeric Go-proteins acting as immediate transducer [224-227]. One of the Go subunits, Gαo, uses an RGS domain to interact directly with Axin, recruiting it to the membrane [54, 228]. Gαo also interacts with Rab5, an interaction that presumuably promotes the internalization of the Wnt/Frizzled/LRP complexes [54, 229]. The Go subunit, Gβγ, recruits Dishevelled to the plasma membrane upon Wnt binding to the Frizzled receptors [54]. 

Together with Axin, two kinases - GSK3 and the primer kinases CK1(α, γ, ε) - are juxtaposed with LRP5/6 [230]. CK1-mediated phosphorylation acts as primer, which triggers GSK3-mediated phosphorylation. CK1γ and GSK3 phosphorylate PP(S/T)PX(S/T) repeats in the cytoplasmic C-domain of LRP5/6, a step that is crucial for rescuing β-catenin from degradation [53, 217, 219, 231]. Upon binding to LRP5/6, the kinases GSK3β and CK1γ switch from phosphorylating β-catenin to phosphorylating LRP5/6 [217, 232]. One model suggested that phosphorylation of Dishevelled-2 by CK1ε increases its affinity to Frizzled receptors [219, 233]. CK1ε was also shown to be required for Dvl-2 phosphorylation and its binding to LRP5/6 [230]. In turn, CK1ε was shown to be directly activated by Wnt signaling through C-terminal dephosphorylation [234].

Recently, the seven-pass transmembrane protein TMEM198 was identified in *Xenopus tropicalis* and shown to associate with LRP6, recruiting CK1 to the receptor complex and promoting LRP6 phosphorylation [235]. Proline-directed kinases have also been shown to be involved in LRP5/6 C-terminal phosphorylation including PKA, Pftk (Cdk14), MAPK (such as p38, ERK1/2, and JNK1) and G-protein-coupled receptor kinases (Grk5/6) [236, 237]. Recently it was also found that Wnt/β-catenin signaling cooperates with tyrosine signaling through FGFR2 (FGF receptor 2), FGFR3 (FGF receptor 3), EGFR (epidermal growth factor receptor) and TRKA kinases (Tyrosine kinase receptor type 1) [238]. Intriguingly, phosphorylated PP(S/T)PX(S/T) peptides alone, derived from the C-terminus of LRP5/6, are able to activate Wnt signaling through a direct inhibition of GSK3 [222, 239].

The release of β-catenin from phosphorylation by CK1α and GSK3 may not be the only mechanism for the Wnt signalosome to regulate β-catenin levels. It was found that LRP6 can stabilize β-catenin indirectly through Axin degradation and GSK3 inhibition [231, 240]. Without the structural protein Axin, CK1α and GSK3 cannot form a complex that phosphorylates β-catenin at the N-terminal end. 

A further mechanism, by which the Wnt signalosome reduces β-catenin degradation, is an induced GSK3 internalization by multi-vesicular endosomes. This physically reduces the cytoplasmic presence of the kinase [241, 242, 243].

The LGR4, -5 and -6 G-protein coupled receptors were shown to associate with the Frizzled-LRP5/6 signalosome and mediate Wnt/β-catenin signaling in intestinal crypt cells. LGR receptors were previously considered to be orphan, but recently R-spondin was identified as their ligand [244, 245, 246].

Furthermore, the parathyroid hormone receptor was found to directly regulate β-catenin signaling through interactions with Dishevelled, but without an involvement of Frizzled receptors [247].

Most of the described receptors are expressed specifically in certain organs, or tissues. Thus parathyroid hormone receptors exert their function in kidneys and bones [248], while LGR4-6 are found in the stomach, in the stem cell compartment of the small intestine and in hair follicles [249]. The complex interface between various receptors and components of β-catenin signaling appear to allow an intricate adaptive regulation.

The Wnt signalosome has been a target for developing antibodies and small drug therapeutics. A monoclonal antibody against Wnt-1 has shown to induce apoptosis in cancer cell lines expressing the Wnt-1 protein [250]. Antibodies against Frizzled-5, developed by OncoMed, have shown anti-tumor properties [251]. The OMP-18R5 antibody, developed in collaboration between Bayer and OncoMed, has entered Phase I clinical trials. Furthermore, antibodies against Frizzled 10 (FZD10) may reduce osteosarcoma growth and metastasis [252]. 

A small molecule, which triggers the internalization of Wnt receptors, has been identified as the FDA approved antihelminthic drug Niclosamide (Table **1**). Amongst other functions, Niclosamide was found to inhibit Wnt/Frizzled-1 signaling with an IC_50_ of 0.5 ± 0.05 μM [253-255]. It also downregulates Dishevelled-2 (Dvl-2) [256] and induces LRP6 degradation in prostate and breast cancer cells [254]. Interestingly, Niclosamide has no reported toxicity against non-cancer cells [256]. 

### Dishevelled

Dishevelled participates in both β-catenin dependent and independent Wnt signaling. Three different Dishevelled proteins are known in humans, which have a similar size and domain organization. All Dishevelled proteins share three functional domains: an N-terminal DIX domain (named after Dishevelled and Axin), a central PDZ domain (Postsynaptic density 95, Discs Large, Zonula occludens-1) and a C-terminal DEP domain (Dvl, Egl-10, Pleckstrin).

The DIX domain is responsible for the polymerization of Dishevelled in the Wnt signalosome [257]. The resulting tetramerization of the Frizzled-LRP5/6 signal complex has been shown to be required for the phosphorylation of the cytoplasmic tail of LRP5/6 [258]. The protein Ccd1, which also has a DIX domain, serves as a positive regulator of Wnt signaling by forming heterodimers with the Dishevelled DIX domain [259]. Recently, the ability of Axin to polymerize through its DIX domain was shown to be crucial for its function in the destruction complex, while a binding between the Axin DIX domain and its Dishevelled counterpart abrogates Axin polymerization. Hence, in addition of being important for the Wnt signalosome, heteromer formation through the DIX domain might be important for inhibiting the formation of the destruction complex by Dishevelled [46, 48], [260, 261].

The DEP domain of Dishevelled was suggested to mediate the interaction with membrane lipids [262] and to facilitate the interaction with Frizzleds through direct binding [263]. 

The PDZ domain of Dishevelled interacts with the cytosolic C-terminal tail of Frizzled [264]. This interaction can be counteracted by the Dapper (Dapper1 and Dapper3) proteins, which bind to the Dishevelled PDZ domain to prevent its interactions with Frizzled [216, 265, 266]. Proteins of Dapper family have been shown to promote Dishevelled degradation mediated by lysosomes instead of proteasomes [265, 267, 268]. Interestingly, Dishevelled was shown to promote Wnt5a-induced endocytosis of Frizzled by using the PDZ-domain to interact with the N-terminal region of β-arrestin 2 [269, 270]. Furthermore, Dishevelled 2 was shown to interact with a subunit of the clathrin adaptor protein AP2, micro2-adaptin. The interaction appears to be required for Frizzled 4 internalization [271].

Phosphorylation modulates the activity of Dishevelled in the Wnt signalosome [272]. Three kinases, CK2, PAR1 and CK1δ/ε that respond to Wnt signaling, have been implicated in Dishevelled phosphorylation [273, 274]. For instance in mouse SN4741 neurons, both Wnt5a and Wnt3a have been shown to induce phosphorylation of Dishevelled-2 and Dishevelled-3 [273]. Based on loss-of-function and gain-of-function experiments a model of a stepwise phosphorylation of Dishevelled was suggested. First, Dishevelled is phosphorylated by the CK2/PAR1 kinases and then by CK1 [274]. It was proposed that CK1ε can inactivate Dishevelled through phosphorylation [274].

The roles of Dishevelled in Wnt/β-catenin signaling go beyond stabilizing the Wnt signalosome and destabilizing the degradation complex. In *Xenopus* it has been demonstrated that mutations in the NLS of Dishevelled attenuate Wnt/β-catenin signaling [275]. Dishevelled translocates to the nucleus, where it interacts with the β-catenin/Tcf complex and participates in transcriptional regulation of β-catenin target genes [276, 277]. In the nucleus, Dishevelled can also form a complex with the histone deacetylase Sirtuin 1 (SIRT1), which supports the transcription of Wnt target genes. In accordance, the SIRT1 inhibitor cambinol negatively regulates Wnt signaling (Table **1**) [278]. Sirtuin1 is a member of the sirtuins proteins family and posseses (NAD^+^)-dependent acetyl-lysine deacetylating activity.

Furthermore, Dishevelled proteins also participate in interactions that affect structural rearrangements of the cell [279]. Through its PDZ domain, Dvl-1 was shown to protect microtubules from depolymerization. It was furthermore demonstrated that the stabilization of microtubules by Dvl-1 is enhanced by GSK3 inhibition [280]. Studies in *C. elegans*, *Drosophila* and vertebrates have led to the conclusion that Wnt/β-catenin signaling may regulate the orientation of the mitotic spindle through Dishevelled [281-283].

Finally, autophagy has been proposed to inhibit Wnt signaling through Dishevelled degradation. It has been shown that an ubiquitination of Dishevelled by the Von Hippel-Lindau protein facilitates its binding to p62, which in turn assists an LC3-mediated recruitment of Dishevelled to autophagosomes [276]. In late stages of colon cancer, a negative correlation between Dishevelled expression and autophagy was observed [276]. 

The PDZ domain of Dishevelled has been used to develop small molecule inhibitors for Wnt/β-catenin signaling. A series of synthetic inhibitors were identified by virtual screening, QSAR and computer-based modeling on the basis of Scaffolds A and B (Table **1**) [284-288]. These compounds interact with the groove of PDZ domain, which interacts with the Dapper proteins [216]. Compound J01-017a (Table **1**) is currently the strongest Dishevelled binder, inhibiting Wnt signaling with a Ki of 1.5+/-0.2 µM [288]. Compound NSC668036 [289] imitates a Dapper protein and binds to the PDZ domain of Dishevelled. Compound 3289–8625 (Table **1**) binds to the same pocket as NSC668036 in Dishevelled with a Kd of 10.6+/-1.7 µM [286]. 

### The Roles of Endocytosis in Wnt/β-Catenin Signaling

Endocytosis plays crucial role in most signaling pathways. In Wnt/β-catenin signaling, both clathrin- and caveolin-mediated endocytosis have been described [246, 290]. Clathrin-mediated endocytosis is mediated through vesicles that are coated by the clathrin protein, also referred to as clathrin-coated pits, while caveolin-mediated endocytosis is characterized by membrane proteins called caveolins which participate in the formation of membrane invaginations called caveolae. 

It has been shown that the Wnt signalosome including Frizzled, GSK-3, Dishevelled and AXIN co-localizes with caveolae where the proteins involved in the signalosome are thought to be sequestrated, preventing them from participating in the formation of a destruction complex [217, 243, 246, 290]. It remains unclear whether all Frizzled receptors can be processed through this endocytic pathway [290]. The involvement of caveolin in the endocytosis of LRP6/Frizzled was shown to be amenable to pharmacological inhibition by filipin (Table **1**) [291]. 

Somewhat contradictory data are published on clathrin-mediated endocytosis [292]. For example, in the ventral cuticle of *Drosophila* larvae, clathrin-mediated endocytosis was reported to be required for the removal of the Wingless protein leading to a downregulation of the signal [293]. However, clathrin-mediated endocytosis was also claimed to be required for Wnt/β-catenin signaling as shown by using the endocytosis inhibitors hypertonic sucrose and chlorpromazine in L-cells [164, 242]. Interestingly, one of the important components of clathrin-mediated GPCR endocytosis is the clathrin-associated sorting protein (CLASP) β-arrestin, which was shown to be required for Wnt/β-catenin signaling [294]. 

For example, co-expression of β-arrestin and Dishevelled was shown to induce Wnt/β-catenin signaling [270, 295]. Furthermore, in *Xenopus* embryos it was shown that morpholinos against β-arrestin reduce endogenous β-catenin levels and interrupt induced axis duplication [270]. In this context it was demonstrated that β-arrestin can form a trimeric complex with Axin, and the N-terminus of Dishevelled [270]. It was also suggested that β-arrestin may couple Frizzled receptors to phosphorylated Dishevelled and thus participate in Wnt/β-catenin signal transduction. [290]. 

Blocking of endocytosis resulted in Dvl-2 degradation [269]. Thus, although endocytosis clears the Wnt signalosome from the cellular surface, there is an increasing evidence that clathrin-depending endocytosis is in itself an important process in Wnt/β-catenin signal activation [164, 290, 292]. 

Endocytosis is not a process that is limited to the core Wnt signalosome. Recently, the endocytic adaptor disabled-2 (Dab-2) was shown to selectively recruit LRP6 to clathrin-dependent endocytosis whereby CK2-mediated phosphorylation of Ser1579 in LRP6 promotes its interactions with Dab-2 and the association with clathrin [296]. Clathrin-mediated internalization was also shown for LGR4-mediated β-catenin signaling, a process that could be disrupted by the small molecule clathrin inhibitor monodansyl-cadaverine (MDC)[244]. Curiously, the Wnt inhibitor Dkk1 also triggers an internalization of LRP6 through clathrin-mediated endocytosis [164, 297]. 

Divergent consequences have been reported for the endocytosis of Wnt signalosomes on Wnt/β-catenin [298]. Endocytotic vesicles containing Wnt signalosomes may shuttle to early endosomes (EE) from which the receptor complex may be sequestrated into intraluminal vesicles of multivesicular endosomes (MVEs). These can either be released as exosomes, whereby exocytosis itself can act as a signal transduction mechanism [199, 298], [299], or MVEs may fuse with lysosomes that lead to a degradation of the included proteins [298, 300, 301]. It is unclear to what stage during this process the Wnt signalosome will remain active, however, deactivation of Wingless was shown to occur after it accumulates in multivesicular endosomes which target it further for lysosomal degradation [302]. It was shown that in response to Wnt ligands or LRP6 overexpression, GSK3 in complex with LRP5/6 is delivered to the lumen of MVEs, separating GSK3 from its cytosolic substrates [241, 303]. Two proteins Hrs/Vps27 and Vps4 that are components of the endosomal sorting (ESCRT) machinery have shown to be required for MVE formation and it has been demonstrated that inhibition of Hrs or Vps4 leads to reduced Wnt/β-catenin signaling [241].

Finally, autophagy can negatively regulate Wnt pathway through the degradation of ubiquitinated Dishevelled which aggregates with LC3-mediated autophagosomes [276]. 

### Connections between the Wnt-Frizzled-LRP5/6 signalosome and the E-cadherin adhesion complex

A close interaction between the Wnt signalosome and cell-cell junctions may exist both on a physical and functional level. N-cadherin [304] and E-cadherin [305] were shown to be able to directly associate with Lrp5/6. Binding of Wnt morphogens to the Frizzled-LRP5/6 induces a CK1ε-dependent phosphorylation of both LRP5/6 and E-cadherin [306] inducing a dissociation of LRP5/6 from E-cadherin [230]. Furthermore, Wnt binding to Frizzled-LRP5/6 induces the phosphorylation of CK1ε dependent phosphorylation of p120 (at Ser268 and Ser269) leading to the dissociation of p120 from the E-cadherin complex [230]. This process was shown to be sensitive to the specific CK1δ/ε inhibitor IC261 (Table **1**) [307, 308]. IC261 was shown to bind tubulin and act as an inhibitor of microtubules polymerization [308]. Intriguingly, p120 that has a major cytoplasmic function at the cytoplasmic end of E-cadherin, also interacts with the Wnt signalosome making it an important linker protein [305]. A depletion of p120 prevents interactions between CK1ε and LRP5/6, disrupting LRP5/6 phosphorylation and AXIN recruitment to the LRP5/6 signalosome, leading ultimately to increased β-catenin degradation [305]. An absence of p120 also has been reported to disrupt CK1ε-mediated phosphorylation of Dvl2 [305]. In turn, signaling through the proteins Dishevelled and Frodo regulates the stability of p120 [309].

Evidence has also been presented that E-cadherin phosphorylation by CK1ε in response to Wnt binding to Frizzled-LRP5/6 decreases the affinity of β-catenin to E-cadherin, leading to the release of β-catenin from its complex with E-cadherin. This process can provide an additional increase of the cellular threshold of free β-catenin. Hence, Wnt signaling can trigger synergistically a stabilization of β-catenin, a release of β-catenin from its complex with E-cadherin and a dissociation of p120 from E-cadherin. 

An inhibitor of CK1δ and CK1ε - PF670462 (Table **1**) was reported to be a potent inhibitor of Wnt/β-catenin signaling with an IC_50_ of ∼17 nM [308]. 

### Adherens complexes in the context of β-catenin signaling

Another major location for β-catenin are adhesion complexes that retain a significant cellular β-catenin pool. Indeed, E-cadherin containing adhesion complexes are supposed to be one of the key regulators of the cytoplasmic β-catenin pool [310]. A simple reduction of cellular E-cadherin was shown to be sufficient to increase significantly free cellular β-catenin, and an abrogation of E-cadherin-mediated adhesion can correlate with an increase in the transcription of β-catenin target genes, a phenomenon that is often accompanied with cancer progression and poor prognosis [311-315]. Strikingly, β-catenin can be actively mobilized from adhesion complexes by Wnt-independent signaling pathways.

Adhesion complexes have multiple roles in the cell, including structural functions, protective functions and a role in signaling pathways, including prominently Wnt/β-catenin signaling [316-319]. The central component of adherens and tight junctions are cadherins, a family of single-pass transmembrane Ca^2+^-dependent proteins. Changes in mutual cadherin concentrations are referred to as cadherin switches. Hence, the increase of N-cadherins against E-cadherins is a hallmark of an epithelial to mesenchymal transition (EMT) both in normal development and in metastasis [320-323]. Cadherin switches are crucial for motility, invasiveness, migration and metastasis in cancer cells [324, 325]. In several works a reverse correlation between invasiveness of cancer cells and E-cadherin-mediated adhesion was shown [326, 327]. 

The extracellular N-terminal domain of E-cadherin mediates cell-cell adhesion and consists of five repeats that are stabilized by calcium ions [321, 328]. In the context of β-catenin-mediated signaling, the intracellular domain of E-cadherin is important. It connects, by its juxtamembrane domain (JMD), adhesion complexes to components of the cytoskeleton involving the armadillo proteins β-catenin and plakoglobin, as well as p120 and α-catenin [329, 330]. The clustering of cadherins is regulated by p120 [331, 332]. Binding of p120 stabilizes cadherins and protects them from internalization and degradation [333, 334] and it has been proposed that the concentration of p120 is a direct limiting factor for cadherins pools [335]. Recruitment of p120 to one type of cadherins sequesters it from binding to another type of cadherins [335]. Hence, a knockdown of p120 reduces cadherin levels by facilitating their degradation, a process that can affect cellular β-catenin levels. p120 is also implied in multiple other ways that attenuate Wnt/β-catenin signaling as described below. 

Centrally important for Wnt/β-catenin signaling is the competitive binding between β-catenin and plakoglobin to E-cadherin through the catenin-binding domain (CBD) [336-340]. All 12 armadillo repeats of β-catenin participate in this interaction. Another armadillo protein - α-catenin - facilitates the interactions between E-cadherin and β-catenin, and anchors actin filaments to β-catenin/E-cadherin [15, 341]. Binding between α-catenin and β-catenin occurs through the first two armadillo repeats of β-catenin [342]. Interactions between β-catenin and α-catenin can be inhibited by Tyr142 and Tyr654 phosphorylation of β-catenin through the tyrosine kinases Fer and Fyn [343, 344].

C-terminal phosphorylation of β-catenin attenuates the affinity between β-catenin and E-cadherin and thus can contribute to regulate free cellular β-catenin levels [19, 311]. Hence, phosphorylation of Tyr654 regulates the orientation of the C-terminal tail of β-catenin, changing its position from closed to open. The induced conformational change enables a number of binding proteins to interact with β-catenin [345]. It has been demonstrated that a Tyr654Glu point mutation in β-catenin imitates the negative charge of a phosphorylation and reduces the affinity of β-catenin to cadherins. Tyr654 phosphorylation of β-catenin also enhances Ser675 phosphorylation by protein kinase A (PKA) [346]. Ser675 phosphorylation appears to promote the stability of β-catenin, and assists in its binding to the Creb Binding Protein (CBP) and as a consequence triggers an enhancement of β-catenin-mediated signaling [347], [348]. In addition, protein kinase B (AKT)-mediated Ser552 phosphosylation of β-catenin promotes its induction of transcription through Tcf/Lef [349-351].

The cellular kinase Src (c-Src) phosphorylates amino acids Tyr86 and Tyr654 in the C-terminus and in the last armadillo repeat of β-catenin respectively [345]. This phosphorylation also structurally impairs β-catenin binding to E-cadherin and can lead to increased cellular β-catenin levels [19, 341, 345, 352]. An increase of Src levels leads to a disruption of intercellular adhesion and E-cadherin dysfunction, while an inhibition of Src by small molecules has the opposite effect [353-355]. Hence, the Src kinase inhibitor bosutinib (SKI-606) (Table **1**) was shown to increase the membrane localization of β-catenin and intercellular adhesion [356-357] and bosutinib has shown promising results in Phase I clinical trials in advanced solid tumors [358]. 

A further mechanism by which β-catenin levels can be regulated at adhesion complexes is the Presenilin 1 (PS1)/γ-secretase system that can cleave the cytoplasmic domain of E-cadherin. The cleavage can be stimulated by calcium influx and has been reported to lead to a disruption of the E-cadherin–β-catenin complex followed by an increase of cytoplasmic α- and β-catenins [359, 360]. Furthermore, the cleaved cytolpasmic terminal fragment (CTF) of E-cadherin has been demonstrated to sequester free β-catenin from the cytoplasm by forming a physical complex with β-catenin. This complex may translocate to the nucleus and interfere directly with Tcf/Lef signaling [314, 361, 362]. Increased β-catenin levels in the cytoplasm and high levels of a sequestrated cytoplasmic domain of E-cadherin were found to correlate with malignancy in esophageal squamous cell carcinoma [363]. Furthermore, it has been shown that tumor invasiveness can be correlated to an accumulation of E-cadherin in the nucleus [363, 364]. Accordingly, an overexpression of E-cadherin lacking a transmembrane and/or an extracellular domain was shown to stabilize cytoplasmic β-catenin levels [363]. Also increased levels of metalloproteinases can lead to a cleavage of the cytoplasmic domain of E-cadherin, as has been shown in metastasic prostate cancers [365], [366]. Alterations in E-cadherin, directly affect the anchoring of actin filaments and simultaneously influence signal transduction mediated through β-catenin and p120 [367, 368].

Intriguingly, X-ray structures of β-catenin with its binding partners (www.rcsb.org) along with biochemical data show that the β-catenin binding proteins Tcf/ICAT/APC and APC/E-cadherin cannot bind β-catenin simultaneously [15, 42, 369]. APC and E-cadherin share the conserved sequence SxxxSLSSL that interacts with the armadillo repeats 3 and 4 of β-catenin, while APC, ICAT, Tcf and E-cadherin have a conserved DxθθxΦx_2-7_E motif (θ-hydrophobic, Φ-aromatic), which binds to the armadillo repeats 5-9 of β-catenin [15]. This is important since E-cadherin may compete with APC for binding to β-catenin or plakoglobin in a mutually exclusive manner [95, 370]. 

Interestingly, also EpCAM (Epithelial cell adhesion molecule), one of the first tumor-associated antigens identified, was shown to be a β-catenin dependent signal transducer, and β-catenin is involved in nuclear signaling by EpCAM itself [371]. It has been demonstrated that a proteolytic cleavage of EpCAM by Presenilin 2 releases EpICD, which forms a complex with β-catenin and Tcf/Lef leading to an induction of c-Myc and Cyclin A and E expression [371, 372]. 

Small molecules targeting cadherins have shown to affect cancer metastasis. The synthetic cyclic pentapeptide, ADH-1 (N-Ac-CHAVC-NH2) targets N-cadherins (it imitates the HAVD amino acid sequence of N-cadherin), increases cellular levels of E-cadherin, and has demonstrated efficacy in Phase I clinical trials against melanoma [373, 374]. Small molecules that influence C-terminal phosphorylation and thus mobilization of β-catenin are discussed below.

### Other Transmembrane Receptors Influencing Wnt/β-Catenin Signaling

The hepatocyte growth factor/scatter factor (HGF) is involved in regulating morphogenesis, embryonal development and regenerative processes [375, 376], and an activation of HGF signaling during tumorigenesis can promote proliferation, angiogenesis and motility [377, 378]. c-Met, the tyrosine kinase receptor of HGF has been linked to β-catenin signaling [379, 380] and it has been shown that HGF/c-Met can activate β-catenin signaling independent from Wnt signaling [381] at the site of E-cadherin containing junctions. Binding of HGF to c-Met triggers an autophosphorylation at Tyr1234 and Tyr1235, which in turn mediates a tyrosine kinase-mediated Tyr654 and Tyr670 phosphorylation of β-catenin [382] inducing the dissociation of β-catenin from E-cadherin [352, 383]. Similarly, Tyr142 and Tyr654 phosphorylation by the FLT3/ITD kinase (Fms-like Tyrosine Kinase-3**)** leads to a dissociation of β-catenin from its complex with E-cadherin [344, 384]. Tyr654 phosphorylation is also required for β-catenin binding to Tcf4, adding to the synergistic effect of c-Met-mediated β-catenin phosphorylation [344]. Accordingly, Imatinib (Table **1**), a tyrosine kinase inhibitor, was shown to reduce Wnt/β-catenin signaling [385]. The small molecule PHA665752 (Table **1**), which inhibits c-Met-mediated phosphorylation, has shown to act inhibitory on HGF induced β-catenin signaling [379, 386]. 

Also the Endothelin A receptor (ET(A)R), through Src-dependent EGFR (epidermal growth factor receptor) transactivation, causes a Tyr654 phosphorylation of β-catenin leading to its mobilization from E-cadherin [387]. Moreover, the receptor tyrosine kinases FGFR2, FGFR3, EGFR and TRKA have recently been shown to increase cytoplasmic β-catenin concentrations via a Tyr142 phosphorylation that releases β-catenin from cadherin complexes [238]. 

In addition to mobilizing β-catenin, a C-terminal phosphorylation of β-catenin protects the protein from Ser/Thr phosphorylation in the degradation complex and thus can lead to increased cytoplasmic levels of β-catenin [380], [388]. It has also been shown that HGF could activate β-catenin signaling through inducing a degradation of E-cadherin which again would lead to a mobilization of β-catenin [366]. The matrix metalloproteinase-7 (MMP-7), a downstream target of Wnt/β-catenin signaling, participates in HGF-induced degradation of E-cadherins. [366]. Furthermore, HGF signaling may also alter β-catenin thresholds secondarily through regulating Snail leading to a repression of the transcription of E-cadherin which in turn leads to a reduced β-catenin pool at cellular junctions [389, 390]. HGF/c-Met-mediated stabilization of β-catenin has been associated with several types of tumors [391]. The small molecule PHA665752 (Table 1), which inhibits c-Met-mediated phosphorylation, has shown to act inhibitory on HGF induced β-catenin signaling [379, 386]. 

Phosphorylation of E-cadherin by CK1δ at Ser846 also reduces its binding to β-catenin [306]. Interestingly, a phosphorylation of E-cadherin and β-catenin (Thr112 and Thr120 by PKD1) can also lead to the opposite effect: to stimulate β-catenin/E-cadherin complex formation [341, 392, 393]. Accordingly, it has been shown that downregulation of PKD1 is associated with advanced prostate cancers [393]. 

### The β-Catenin Tcf/Lef Transcription Complex

Besides its implications in junctions, the main effector function of β-catenin is in the nucleus. Here it regulates transcription through interactions with a number of transcription factors, including predominantly Tcf/Lef, Hif-1 and possibly also Oct4. β-catenin may also have a more unspecific role on transcription regulation through interactions with chromatin. The shuttling of cytoplasmic β-catenin to the nucleus and back to the cytoplasm is not entirely understood. A picture emerges, where the nuclear uptake of β-catenin can be enhanced by the context-dependent C-terminal phosphorylation of β-catenin at S675 by PKA. Evidence suggests that the export of β-catenin from the nucleus to the cytoplasm can be GSK3-dependent [394]. Both kinases are well-explored drug targets. A further mode of β-catenin transport to the nucleus was proposed to be a binding between β-catenin and Tcf/Lef in the cytoplasm, followed by its transfer to the nucleus [12, 13, 395]. Other mechanisms that influence the shuttling of β-catenin to the nucleus have been discussed earlier.

In the nucleus, the interaction between β-catenin and the zinc finger transcriptional factors of the Tcf/Lef family has been described and is seen as the classical regulatory unit for Wnt/β-catenin target genes (http://www.stanford.edu/~rnusse/wntwindow.html). All members of the Tcf/Lef family (Tcf-1, Tcf-3, Tcf-4 and Lef1) contain an N-terminal binding domain for β-catenin, followed by a context-dependent regulatory domain (CRD) with binding sites for the co-repressor Groucho (Gro), a HMG-box DNA-binding domain, and a C-terminal domain with binding sites for the co-repressor C-terminal binding protein (CtBP) [396, 397]. Lef1 in general acts as a transcriptional activator in complex with β-catenin. Tcf3 is considered to be predominantly a transcriptional repressor. Tcf-1 and Tcf-4 have been claimed to execute context dependent dual activator or repressor roles [396]. In mice, Tcf-3 represses Wnt/β-catenin signaling either through a competitive physical interaction with β-catenin or via competition for Tcf/Lef binding sites on DNA [396, 398]. Further diversity of family members may be created by alternative splicing [399].

In the absence of β-catenin, members of the Tcf/Lef family form a complex with co-repressors such as Groucho, CtBP, and HDAC leading to a repression of the transcription complex [5, 400-402]. β-catenin directly displaces Groucho/TLE from Tcf/Lef by binding to a N-terminal low-affinity binding site that overlaps with the Groucho/TLE-binding site rendering the Tcf/Lef into a transcription activator [403]. It has been reported that the interactions between β-catenin and Tcf/Lef are charge-dependent and occur through the formation of salt bridges between Lys amino acids of β-catenin and Glu amino acids of Tcf [404]. The histone acetyltransferase CREB binding protein (CBP) attenuates the complex and acts as a context-dependent transcriptional regulator [394].

Also, the armadillo protein plakoglobin is able to associate with the Tcf/Lef transcriptional complex, although with less affinity than β-catenin, and early reports claim that both proteins are able to activate Tcf/Lef reporters [405-407]. Plakoglobin was also shown to promote transcriptional activity independently from β-catenin [407], and although plakoglobin was shown to be less potent to activate Wnt/β-catenin downstream genes, c-Myc expression is significantly elevated by plakoglobin [408]. Interestingly, similar to β-catenin, ectopic over-expression of plakoglobin was shown to lead to axis duplication in *Xenopus* [409]. Further indications for a functional redundancy between the two structurally related proteins come from mouse studies showing that mice lacking plakoglobin do not show developmental apparent abnormalities. Furthermore, a decrease of plakoglobin in *Xenopus* does not affect embryonic axis formation [405]. 

p120 also affects Wnt/β-catenin-mediated transcription. In the absence of phosphorylated p120, the zinc finger transcription factor Kaiso binds to the HMG domain of Tcf, forming a co-repressor complex together with histone deacetylase (HDAC). Phosphorylation of p120 causes its dissociation from E-cadherin, its entrance to the nucleus and binding to Kaiso. In consequence, Kaiso loses its role as a co-repressor [230]. Kaiso binding sites are frequently located near Wnt responsive elements of several β-catenin target genes including Siamois, c-Myc and cyclin D1 [410-413]. Notably, this process was shown to be enhanced by Wnt-signaling indicating that several armadillo components that are present in adhesion junctions could be involved in mediating a convergent signaling program. 

The Tcf/Lef transcriptional complex has a multitude of further binding partners [402]. Proteins like Pontin52 [414], the TATA-binding protein [415], Bcl-9/Legless, and Pygopus [416, 417] have all shown to promote the formation of a β-catenin/Tcf complex. Chibby (Cby), a small (126 aa) protein antagonizes Wnt/β-catenin signaling by forming a ternary complex with protein 14-3-3ζ and β-catenin [418, 419]. The protein TC-1, associated with thyroid cancer, in turn can bind to Cby and inhibit its interactions with β-catenin, leading to an upregulation of β-catenin target genes [420, 421]. Further tissue-specific proteins like Osterix (osteoblasts-specific transcription factor) are also able to repress the transcriptional complex through a disruption of Tcf binding to DNA [422]. As earlier described, Dishevelled in response to Wnt signaling may also localize to the nucleus [275, 423] where it forms a quaternary functional complex with Tcf/Lef and c-Jun, whereby c-Jun acts as scaffold [277]. Further proteins, associated with the Tcf/Lef complex and regulate its activity are reviewed in [396, 402].

Tcf/Lef-mediated transcription is target of numerous regulative covalent modifications like phosphorylation, SUMOylation, ubiquitination, and acetylation [201, 396]. It has been shown that CK1δ and CK2 phosphorylate Lef-1 [424] leading to a disruption of interactions between β-catenin and Lef-1, but not between Lef-1 and the template DNA. Hence, CK1δ-mediated phosphorylation results in a transcriptional repression of Lef-1/β-catenin target genes. In contrast, it has been demonstrated that Ser42 and Ser61 phosphorylation of Lef-1 by CK2 enhances Lef-1/β-catenin-mediated transcription [424, 425]. Initially it was supposed that CK2-mediated phosphorylation increases Lef-1 affinity to β-catenin. However, affinity studies have shown that Lef-1 phosphorylation does not affect its binding to β-catenin [426]. Instead, it was found that CK2-mediated phosphorylation leads to a decrease of Lef-1 interactions with the Gro/TLE1 co-repressor [427]. 

The Nemo-like kinase (NLK) phosphorylates amino acids Thr155 and Ser166 of Lef-1 and amino acids Thr178 and Thr189 of Tcf-4 which have been shown to lead to a reduced DNA-binding [20, 428, 429]. Thus, NLK has been proposed to be a negative regulator of β-catenin/Tcf controlled transcription [430]. Indeed, in human embryonic kidney 293 (HEK293) cells and the cervical epithelioid carcinoma cell line HeLa, NLK inhibits β-catenin-regulated target genes expression [428, 431]. Curiously, in zebrafish midbrain and mammalian neural progenitor cell (NPC)-like cell lines, NLK-mediated phosphorylation of Lef-1 upregulates Wnt signaling [432]. 

The E3 ligase PIASy SUMOylates Lef-1, which could lead - context dependent - to either an activation or to an inhibition of Lef-1 [72, 433]. 

Different small molecule inhibitors that act at the level of the Tcf/Lef transcription complex have been reported. ICG-001 selectively binds to CBP, but not to the closely-related protein p300. ICG-011 disrupts the interaction of CBP with β-catenin and downregulates target genes expression [434]. Recently, ICG-001 has shown to be able to block EMT (epithelial to mesenchymal transition) induced by TGFβ1 in a RLE-6TN rat lung epithelial-T-antigen negative cell line. [435]. ICG-001 has reached Phase 1 clinical trials [436]. During a high-throughput screening the approved FDA diuretic ethacrynic acid (Table 1) was found to down-regulate Wnt/β-catenin signaling by inhibiting the formation of the β-catenin/Lef-1 complex [437, 438] in chronic lymphocytic leukemia (CLL) cells, although at a low IC_50_ (Table 1). A number of ethacrynic acid derivatives have since been synthesized leading to a significant potency improvement [439]. 

Several small molecules were found among natural products that disrupt the β-catenin/Tcf-4complex: CGP049090, PKF118-310, PKF115-584 and ZTM000990, all with an IC_50_ slightly below 1 µM (Table 1) [440-442]. Docking studies have shown that the assumed binding site for these compounds in β-catenin corresponds to a cavity located between amino acids Arg469, Lys435, Lys508, Glu571 and Arg515 which interacts with Tcf-4 [443]. A further set of compounds, PNU-74654 and BC21 (Table 1), also inhibits the interactions between β-catenin and Tcf. The binding of BC21 to β-catenin was shown to depend on the polar amino acids Lys435, Arg469, Lys508, Arg515, and Glu571 of β-catenin [443, 444].

In addition to Tcf/Lef, the oxygen sensing zinc finger transcription factor Hif-1α has been pointed out as a central regulatory element in β-catenin signaling. In an oxygen rich environment, Hif-1α gets hydroxylized through the HIF prolyl hydroxylase, triggering a subsequent ubiquitination by the von Hippel-Lindau protein (pVHL) that targets Hif-1α for rapid degradation in the proteasome [445]. Strikingly, the von Hippel Lindau protein has also been implied in promoting the degradation of cytoplasmic β-catenin, while maintaining the expression of E-cadherin [446]. In contrast, it was shown that PI3K (Phosphoinositide 3-kinase) through MAPK induces Hif-1α signaling [447]. A second positive regulatory pathway has been described for PI3K/mTOR that is involved in regulating Hif-1α protein synthesis through AKT/PKB (protein kinase B), mTOR (mammalian target of rapamycin) and S6K (p70 S6 kinase) [448]. It has recently been demonstrated that the PI3K inhibitor GDC-0941 represses Hif1α and Hif-2α expression and activity [449]. 

In a low oxygen environment – as frequently found in stem cell niches – Hif-1α forms a complex with ARNT (the constitutive active form of Hif-1α) and enters the nucleus where it competes with Tcf/Lef proteins for β-catenin binding [450]. Hence, it has been proposed that while under normoxic conditions β-catenin binds predominantly to Tcf/Lef and activates classical Wnt/β-catenin downstream genes, under hypoxic conditions, Hif-1α may recruit β-catenin to alternative binding sites at promoters e.g. promoters that enhance tumor survival [446]. Strikingly, the promoters of genes of Tcf-1 and Lef-1contain hypoxia response elements (HREs) [451] and Hif-1α is directly involved in regulating Tcf/Lef protein abundance [451]. Hif-1α also is involved in regulating the transcription of proteins implied the destruction complex, and has been shown to negatively regulate the transcription of APC via hypoxia-responsive elements, which could lead to increased cellular β-catenin levels. In a feedback loop APC mediates a repression of Hif-1α while APC depletions result in increased Hif-1α levels [452]. Furthermore, GSK3 has been reported to phosphorylate and destabilize Hif-1α [132].

### Cross-talk with other Pathways: Interconnections Between Inflammation and Wnt/β-Catenin Signaling

Both COX activity and Wnt/β-catenin signaling are interconnected and have been associated with tumorigenesis. An inhibition of the cyclooxygenases COX-1 and COX-2 leads to reduction of prostaglandin synthesis including the pro-inflammantory prostaglandin E2 (PGE2) [453]. PGE2 activates a signaling cascade through the EP2 and EP4 receptors that leads to a PKA dependent Ser552 and Ser675 phosphorylation of β-catenin. The phosphorylation promotes β-catenin stabilization and nuclear uptake [52, 347, 348]. Strikingly, it was shown that Tyr654 phosphorylation of β-catenin, e.g. by receptor tyrosine kinases [311, 454], facilitates a Ser675 phosphorylation by PKA [346]. Hence, a model has been proposed whereby an activation of c-Met induces Tyr654 phosphorylation of β-catenin, which leads to a dissociation of β-catenin from adhesion complexes followed by a possible phosphorylation by PKA on Ser674, which enhances nuclear uptake of β-catenin and a recruitment of transcription factors [346]. In addition, it was shown that PGE2 interaction with EP1-4 receptors leads to activation of Gα_s_ which competes with APC for binding to the RGS domain of Axin [455]. Hence, PGE2 may also contribute to a Wnt-independent destabilization of the β-catenin destruction complex [455]. Finally, PKA-mediated phosphorylation of GSK3 may also lead to increased β-catenin levels [456].

Recently a connection between prostaglandin H2 and Wnt/β-catenin signaling was shown on the transcriptional level. Hence, the gene encoding 15-prostaglandin dehydrogenase, which dehydrogenates prostaglandin H2, was shown to be repressed by β-catenin [457]. Furthermore, mutated APC can lead to an elevated COX2 gene expression [458].

A number of anti-inflammatory COX-2 inhibitors were reported to affect Wnt signaling [459]. In this context, it has been demonstrated that an inhibition of COX enzymes by non-steroid anti-inflammatory drugs or aspirin can reduce the risk of Wnt/β-catenin dependent colorectal cancers significantly [460]. Furthermore, it was shown that Rp-8-Br-cAMP (Table 1), a small molecule inhibitor of PKA, reduces the translocation of β-catenin to the nucleus and reduces the expression of Wnt/β-catenin target genes [461].

### TGFβ and β-Catenin Signaling

The TGFβ signaling pathway belonging to the same protein superfamily as bone morphogenic proteins (BMP) and Nodal, is involved in multiple biological processes including proliferation, apoptosis and cancerogenesis [462-464]. TGFβ and Wnt/β-catenin signaling are interconnected at several levels through Smad proteins. In particular the TGFβ inhibitory protein Smad7 is regulated by components of the Wnt/β-catenin signaling pathway but it also affects Wnt/β-catenin signaling[464, 465]. For instance it has been shown that Axin assists in the degradation of Smad7 through serving as a scaffold for the E3 ligase Arkadia, which ubiquitinates Smad7 and targets it for degradation [464]. Also the ubiquitin ligase Smurf2, which belongs to the HECT class of ubiquitin ligases, binds Smad7 and targets the TGFβ receptors for degradation [466]. Interestingly, Smurf2 has also been suggested to act as as an ubiquitin ligase for Axin, and a knockdown of Smurf2 leads to a reduction of β-catenin/Tcf reporter activity [467]. Furthermore, it was shown in mouse keratinocytes that Smad7 associates with β-catenin and enhances its degradation by recruiting the E3 ubiquitin ligase Smurf2 [468]. Hence, a knockdown of Smad7 leads to an increase of β-catenin-mediated signaling [468]. Smad7 was also shown to interact with the β-catenin-Tcf/Lef transcriptional complex and to regulate apoptosis in a TGFβ dependent manner [469]. It has been proposed that Smad7 selectively downregulates the mobile pool of β-catenin while it upregulates the pool of β-catenin that interacts with E-cadherin [468, 470, 471].

Smad7 is not the only representative of the Smad family that affects β-catenin. Smad4 in complex with its receptor R-Smad interacts with β-catenin in the nucleus. In chondrocytes it was shown that the C-terminal domain of Smad3 interacts with the N-terminal and central domains of β-catenin in a TGF-β-dependent manner [81].

Finally, the TGFβ pathway was shown to induce phosphorylation of β-catenin at Tyr654 through an activation of Src kinase(s), influencing both the presence in junctional complexes, and the nuclear localization of β-catenin [163]. 

### Interconnections Between PDGF and β-Catenin Signaling

Platelet-derived growth factor (PDGF) regulates cellular division and participates in angiogenesis. PDGF treatment leads to a phosphorylation of the p68 helicase, which facilitates the nuclear translocation of β-catenin and its interaction with the Tcf/Lef complex [472]. Interestingly, in a prostate cancer model, PDGF has shown to promote the formation of a nuclear transcription complex including β-catenin and Hif-1α, establishing a link between PDGF signaling, hypoxia and β-catenin [473]. In an apparent feedback loop, the extracellular Wnt inhibitor sFRP1 was shown to increase the expression of platelet-derived growth factor-BB (PDGF-BB) in mesenchymal stem cells (MSC) [474].

### Interconnections Between Notch and β-Catenin Signaling

Both Notch and Wnt/β-catenin signaling are interconnected. Recently it was shown that membrane-associated uncleaved Notch directly interacts with β-catenin, serving as a protein trap and down-regulating the cellular levels of β-catenin. This process has been demonstrated to require the endocytic adaptor protein Numb and lysosomal activity [475]. In turn, the Notch ligand Jag1, which is a target of Wnt/β-catenin signaling, functions as a Wnt-dependent Notch activator [476]. 

## CONCLUDING REMARKS

Although Wnt/β-catenin is a major signaling pathway with very significant implications in a broad range of diseases, addressing the pathway through small drugs or therapeutic antibodies is still at its infancy. Despite the description of a multitude of interesting bio-targets in the pathway, together with the identification of reagents that interfere with these bio-targets, it is by no means clear which bio-targets in the pathway may give a lead in drug discovery.

Creating specific agents to any bio-target is a challenge. In addition, several of the known bio-targets in the Wnt/β-catenin pathway are also implied in a multitude of other pathways raising specificity issues. Since the Wnt/β-catenin signaling pathway is highly complex, a number of back-up mechanisms as well as feedback loops exist. In this context it is also necessary to bear in mind that β-catenin is a member of a larger protein family – the armadillo protein family - and other members of that family, including p120 and plakoglobin, may have functions that are supportive to, or overlapping with β-catenin. Although it is not settled where the best druggable bottlenecks in the pathway may be, or if such bottlenecks exist at all, three main interference points in the pathway are at current predominantly explored: (i) the Wnt signalosome, (ii) the destruction complex and (iii) β-catenin targets and interactions in the nucleus. In a broader sense, other manipulations including altering cellular junctions, influencing prostaglandine-mediated signaling, affecting HGF signaling and mechanisms that influence Hif-1α levels are important in the context of addressing Wnt/β-catenin signaling. 

Hence, it is not clear to what extent the pathway could be silenced by a single therapeutic agent and whether it may ultimately be necessary to regulate the pathway at two or multiple points simultaneously in an approach that may be termed “cloud inhibition”. Indeed, a combination of an EGFR and Tankyrase inhibition recently revealed a close functional correlation of both pathways and confirmed the synergistic effect of a dual antagonistic treatment in lung cancer cells [477]. Zibotentan, a ET(A)R antagonist when combined with the EGFR inhibitor gefitinib, reduces β-catenin activity [387]. Furthermore, it has been shown that β-catenin-mediated resistance to PI3K and AKT inhibitors can be reversed by XAV939 (Table **1**), a PARP/Tankyrase blocker [478]. 

However, even if Wnt/β-catenin signaling can be reduced more stringently with combinations of inhibitors, it is important to note that a complete inhibition of Wnt/β-catenin signaling may not be desirable as it may be necessary to maintain a basic Wnt/β-catenin activity in an organism to ensure the viability of natural Wnt dependent cells.

Despite of all the described issues, significant excitement is sensed in the field that developing reagents altering Wnt/β-catenin signaling can provide valuable new therapeutic tools which will allow to address disease conditions that have hitherto escaped therapeutic success.

## Figures and Tables

**Fig. (1):**
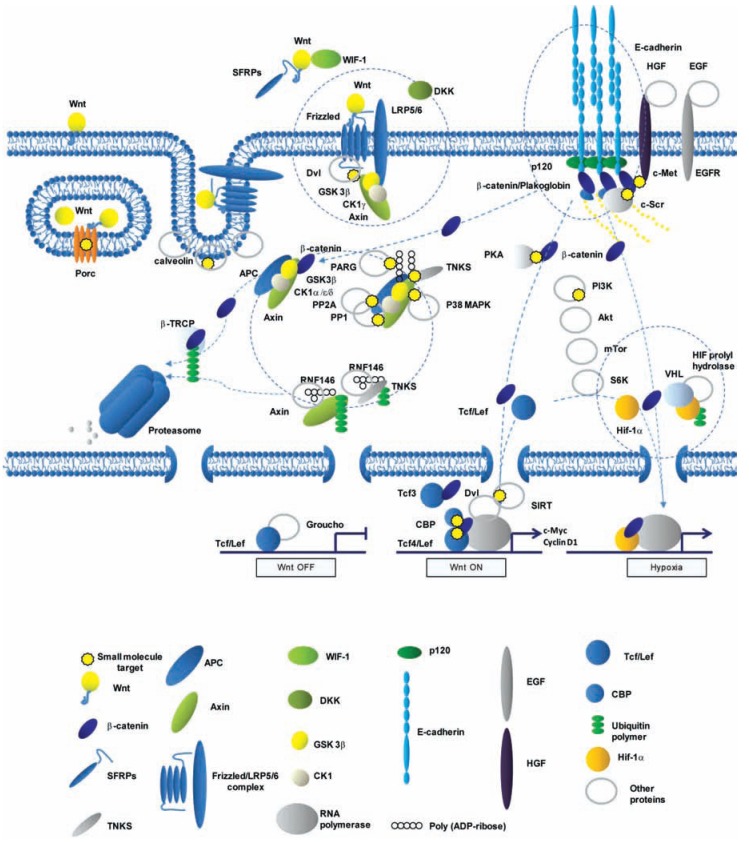
Simplified schematic representation of drug targets (yellow stars) in Wnt/β-catenin-mediated signaling. Four key aspects that regulate β-catenin-mediated signaling are highlighted: the destruction complex, the Wnt/β-catenin signalosome, cadherin junctions, and the hypoxia sensing system Hif-1α (hipoxia induced factor 1β). Proteins that directly interact with Wnt/β-catenin are marked as colored structures, other proteins are marked as circles.

**Table 1.** Small Molecules, which Downregulate Wnt/β-Catenin Signaling

Structure	Compound	Target	Reference
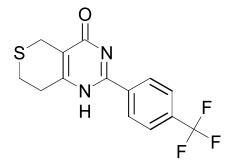	XAV939	Tankyrases1, 2	[37]
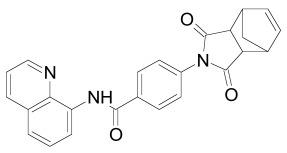	IWR1	Tankyrases1, 2	[38]
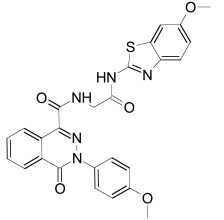	IWP-1	Porcupine	[38]
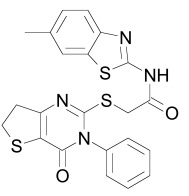	IWP-2	Porcupine	[38]
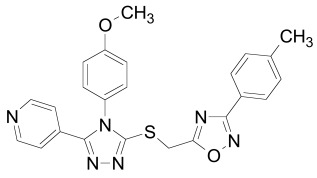	JW74	Tankyrases1, 2	[39]
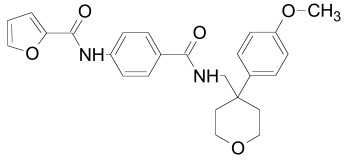	JW55	Tankyrases1, 2	[40]
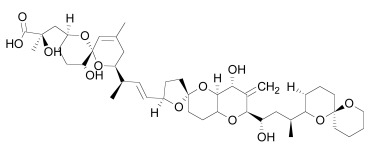	Okadaic acid	PP2A phosphatase	[58]
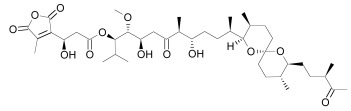	Tautomycin	PP1 phosphatase	[60]
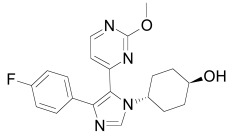	SB239063	p38 MAPK	[122, 137]
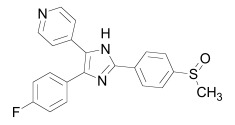	SB203580	p38 MAPK	[122, 137]
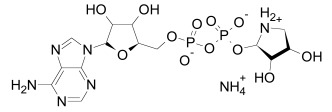	ADP-HPD	PARG	[148]
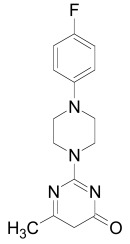	2-[4-(4-fluorophenyl)piperazin-1-yl]-6-methylpyrimidin-4(3H)-one	Tankyrases1, 2	[165]
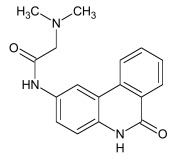	PJ34	Tankyrases1, 2	[170]
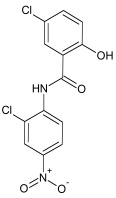	Niclosamide	Downregulates Dvl-2, triggers LRP6 degradation	[253, 254, 255]
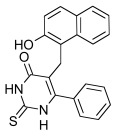	Cambinol	SIRT1	[278]
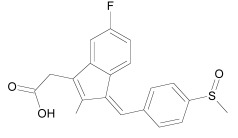	Sulindac	PDZ domain of Dishevelled	[284, 479]
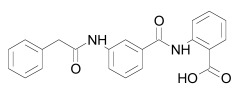	3289-8625	Dishevelled	[286]
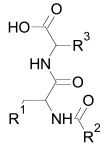	Scaffold A for series of analogs	Dishevelled	[288]
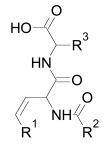	Scaffold B for series of analogs	Dishevelled	[288]
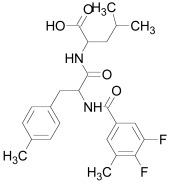	J01-017a	Dishevelled	[288]
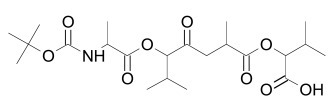	NSC668036	Dishevelled	[289, 441]
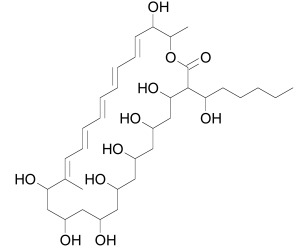	Filipin	Caveolin-mediated endocytosis	[291]
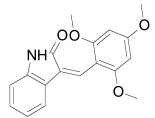	IC261	CK1ε/δ	[308]
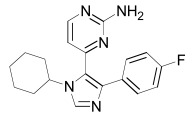	PF670462	CK1δ and CK1ε	[308]
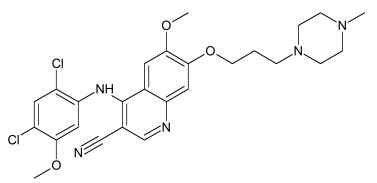	Bosutinib	Src kinase	[356, 357]
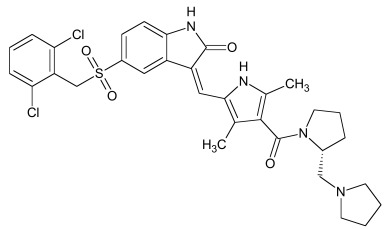	PHA665752	c-Met	[379, 386]
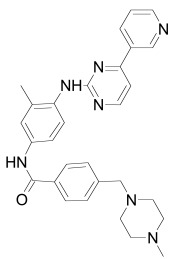	Imatinib	Different tyrosine kinases	[385]
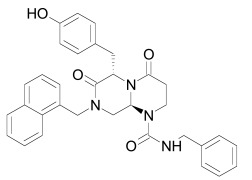	ICG-001	CREB binding protein (CBP)	[434, 441]
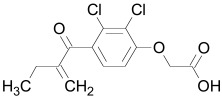	Ethacrynic acid	Lef-1	[437, 439]
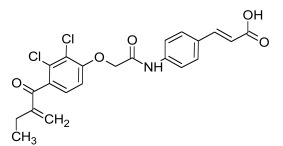	Ethacrynic acid derivative	Lef-1	[439]
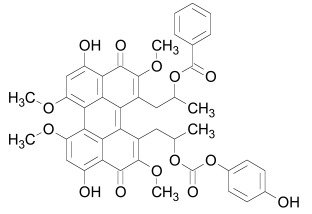	PKF115-584	β-catenin	[440, 441, 443]
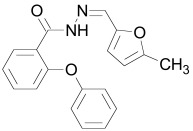	PNU-74654	β-catenin	[441, 443]
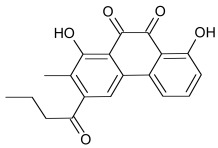	PKF118-744	β-catenin	[440, 441, 443]
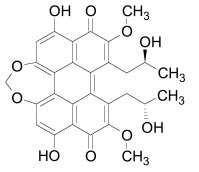	CGP049090	β-catenin	[440, 441, 443]
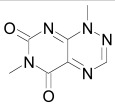	PKF118-310	β-catenin	[440, 441, 443]
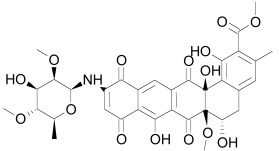	ZTM000990	β-catenin	[440, 441, 443]
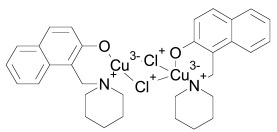	BC21	β-catenin	[444]
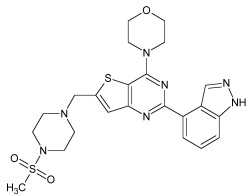	GDC-0941	PI3K	[449]
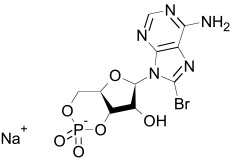	Rp-8-Br-cAMP	PKA	[461]

## ABBREVIATIONS

ADP-HPD: = Adenosine 5'-diphosphate (hydroxymethyl) pyrrolidinediol
ADH-1: = Peptide N-Ac-CHAVC-NH2
APC: = Adenomatosis polyposis coli
AP-1: = Activator protein 1 (AP-1), transcription factor
AP-2alpha: = Transcription factor activator protein
Micro2-adaptin: = Subunit of clathrin AP2 adaptor protein
ADP: = Adenosine diphosphate
Axam: = Axin associating protein
Axin2: = Axis inhibition protein 2
β-TrCP: = β-transducin-repeat-containing protein
Bcl-9: = B-cell CLL/lymphoma 9 protein
B56α: = Protein Phosphatase 2A subunit
CBD: = Catenin-binding domain
CBP: = Protein cAMP response element-binding (CREB)-binding protein
Ccd1: = Coiled-coil-DIX1
Cer: = Cerberus
c-Jun: = Transcription co-factor
Diversin: = Planar cell polarity protein, containing ankyrin domain
DIX domain: = Dishevelled/Axin homologous domain
CK2: = Caseine kinase 2
CDK1: = Cyclin-dependent kinase 1
CLASP: = Clathrin-associated sorting protein
CK1α: = Casein kinase 1α
CK1γ: = Casein kinase 1γ
CK1δ: = Casein kinase 1δ
CK1ε: = Casein kinase 1ε
c-Met: = Hepatocyte growth factor receptor (HGFR)
c-Myc: = Myelocytomatosis virus oncogene cellular homolog
CRD: = Context-dependent regulatory domain in proteins of Tcf/Lef family
CREB: = Cyclic-AMP response element-binding protein
CtBP: = C-terminal binding protein
CTF: = Cytolpasmic terminal fragment
Dab-2: = Endocytic adaptor disabled-2
Dapper: = Dishevelled-associated antagonist of β-catenin
DEP domain: = Dvl, Egl-10, Pleckstrin
DIX domain: = Dishevelled/Axin homologous domain
DKK: = Dickkopf
Dvl/Dsh: = Dishevelled
EE: = Early endosomes
EB1: = Microtubule-associated protein RP/EB family member 1
EBNA-1: = Epstein-Barr nuclear antigen 1
EGFR: = Epidermal growth factor receptor
EMT: = Epithelial-mesenchymal transition
EpCAM: = Epithelial cell adhesion molecule
ESCRT: = Endosomal sorting complex required for transport
ET(A)R: = Endothelin A receptor
Ex vivo: = Experiments conducted with tissues outside of the organism, but in conditions close to natural
Evi/Wls: = Evenness/Wntless
FDA: = Food and drug administration
Fer: = Proto-oncogene protein tyrosine kinase
FGF: = Fibroblast growth factor
FGFR2: = Fibroblast growth factor receptor 2
FGFR3: = Fibroblast growth factor receptor 3
FLT3/ITD: = Cluster of differentiation antigen 135 (CD135), also known as Fms-like tyrosine kinase 3
FRAT1: = Frequently rearranged in advanced T-cell lymphomas 1 proto-oncogene
FRAT2: = Frequently rearranged in advanced T-cell lymphomas 2 proto-oncogene
FZ, FZD: = Frizzled, receptor of Wnt proteins
Frodo: = Signaling adaptor protein
FYN: = Proto-oncogene protein tyrosine kinase
Gα, Gβ, Gγ, Go: = Guanine nucleotide binding proteins
GPI: = Glycophosphatidylinositol
GLUT4: = Glucose transporter type 4
GPCR: = G-protein coupled receptor
G proteins: = Guanine nucleotide-binding proteins
Grk5/6: = G-protein-coupled receptor kinases
GRB14: = Growth factor receptor-bound protein 14
GPR177: = G protein-coupled receptor 177 (GPR177)
GSK3: = Glycogen synthase kinase 3
GTPase: = GTPase-activating protein
hDLG1: = Human disks large homolog 1
HECT: = Domain homologous to the E6-AP carboxyl terminus
Hif-1α: = Hypoxia-inducible factor 1α
HGF: = Hepatocyte growth factor
HGFR: = Hepatocyte growth factor receptor
HPS domain: = His/Pro/Ser domain of tankyrase
HRS/VPS27: = Hepatocyte growth factor receptor tyrosine kinase substrate
HSPG: = Heparane sulfate proteoglycans
HUWE1: = HECT-type ubiquitin E3 ligase 1
IC_50_: = Half maximal (50%) inhibitory concentration (IC) of a substance
ICAT: = Inhibitor of β-catenin and Tcf4
I-MFA: = Inhibitor of myogenic basic helix-loop-helix transcription factors
IRAP: = Insulin-responsive amino peptidase
IWP: = Inhibitor of Wnt production, small molecule
IWR: = Inhibitor of Wnt response, small molecule
Jag1: = Jagged 1 protein
JMD: = Juxtamembrane domain
JNK: = c-Jun N-terminal kinase
JW74: = Inhibitor of tankyrases, small molecule
Kaiso: = Bi-modal DNA-binding protein, transcription regulator
Kd: = Dissociation constant
Ki: = Inhibition constant
LC3: = Light chain of the microtubule-associated protein 1
LEF: = Lymphoid enhancer factor-1; transcription factor
LGR4, 5: = Leucine-rich repeat-containing G protein-coupled receptor 4, 5
LRP5/6: = Low-density lipoprotein receptor-related proteins 5/6
MAPK: = Mitogen-activated protein kinase
Mcl-1: = Myeloid cell leukaemia 1
MDC: = Monodansyl-cadaverine
MDCK cells: = Madin-Darby canine kidney (MDCK) cells
MEKK1: = MAP kinase kinase kinase 1
MEKK4: = MAP kinase kinase kinase 4
MM: = Multiple myeloma
MMP-7: = Matrix metalloproteinase-7
MSC: = Mesenchymal stem cells
MVEs: = Multivesicular endosomes
NLS: = Nuclear localization signal sequences
NAD^+^: = Nicotinamide adenine dinucleotide
NLK: = Nemo-like kinase
NuMa: = Nuclear mitotic apparatus protein 1
Oct-4: = Octamer-binding transcription factor 4
OMP-18R5: = OncoMed Pharmaceuticals 18R5
Osterix: = Osteoblast specific transcription factor
PAR1: = Prader-Willi/Angelman region-1
PARG: = Poly (ADP-ribose) glycohydrolase
PARP: = Poly (ADP-ribose) polymerase
PCNA: = Proliferating cell nuclear antigen
p62: = Nucleoporin 62, a protein complex associated with the nuclear envelope
PDB: = Protein Data Bank, www.rcsb.org
PDGF: = Platelet-derived growth factor
PDZ domain: = Postsynaptic density 95, Discs Large, Zonula occludens-1
PFTK (CDK14): = Serine/threonine-protein kinase PFTAIRE-1
PGAP1: = Glycosylphosphatidylinositol (GPI)-inositol-deacylase
PGE2: = Prostaglandine E2
PIAS: = Protein inhibitor of activated STAT-1, E3 SUMO-protein ligase
PI3K, PI4KII, PIP5KI: = Phosphatidylinositol kinases
PKA: = Protein kinase A
PKB (AKT): = Protein kinase B
PKC: = Protein kinase C
PKD1: = Protein kinase D1
PLK1: = Serine/threonine-protein kinase, also known as polo-like kinase 1
PORC: = Porcupine
PP1: = Protein phosphatase 1
PP2A: = Protein phosphatase 2A
PP2C: = Protein phosphatase 2C
PRMT1: = Arginine methyltransferase
PS1: = Presenelin 1
PTP-BL: = Protein tyrosine phosphatase
p38 MAPK: = p38 mitogen-activated protein kinase
p70S6K: = p70 ribosomal S6 kinase
p90RSK/MAPKAP: = p90 ribosomal S6 kinase/MAPK-activating protein
p120: = Armadillo repeats containing protein, referred to also as catenin δ
QSAR: = Quantitative structure-affinity relashionship
Rab5: = Ras superfamily of monomeric G proteins, localized to clathrin vesicles and early endosomes
RGS domain: = Regulators of G protein signaling
RNF146: = RING finger protein 146 ubiquitin ligase
R-Smad: = Receptor of Smad 4
R-spondin: = Secreted activator protein
SAM domain: = Sterile alpha motif
SAR: = Structure-activity relationship
SFRP: = Soluble Frizzled-related protein
SIRT1: = Histone deacetylase Sirtuin 1
SN4741: = Dopaminergic neurons derived from transgenic mouse embryos
Smad3: = Mothers against devapentaplegic 3
Smad4: = Mothers against devapentaplegic 4
Smad5: = Mothers against devapentaplegic 5
SNAIL: = Zinc-finger transcription factor
c-SRC: = Cytoplasmic SRC tyrosine kinase (Src - abbreviation of sarcoma)
STF: = Super TOPFlash reporter containing Tcf/Lef binding sites
SUMO: = Small Ubiquitin-like Modifier protein
SUMOylation: = Post-translational modification of proteins by adding SUMO
TAB182: = Tankyrase 1 binding protein 182
TATA-binding protein: = Transcription factor, which binds TATA DNA sequence
Tankyrase: = TRF-1-interacting ankyrin related ADP-ribose polymerase
TC-1: = Thyroid cancer protein 1
TCF: = T- cell factor; transcription factor
TERT: = Telomerase reverse transcriptase subunit
TGFβ: = Transforming growth factor β
TGN: = Trans-Golgi network
TLE1: = Transducin-like enhancer protein 1
TMEM198: = Transmembrane protein 198
TRF1: = Telomeric repeat binding factor 1
TRKA kinases: = TRK1-transforming tyrosine kinase protein or Trk-A
RNF146: = Poly(ADP-ribose)-directed E3 ligase
ROR: = RAR-related orphan receptor
RYK: = Related to receptor tyrosine kinase
V-ATPase: = Vacuolar-type H+-ATPase
WIF-1: = Wnt inhibitory factor-1
WNT: = Wntless/Int1 signaling protein
WWE domain: = Common interaction module in protein ubiquitination and ADP ribosylation, has 3 conserved amino acids - two tryptophans and glutamate
Xpo1(Exportin 1): = Protein, participating in the nuclear export

## References

[1]  Nusse R, Varmus HE (1982). Many tumors induced by the mouse mammary tumor virus contain a provirus integrated in the same region of the host genome. Cell.

[2]  Baker NE (1987). Molecular cloning of sequences from wingless, a segment
polarity gene in Drosophila: the spatial distribution of a transcript
in embryos. EMBO J.

[3]  McMahon AP, Moon RT (1989). Ectopic expression of the proto-oncogene
int-1 in Xenopus embryos leads to duplication of the embryonic
axis. Cell.

[4]  Logan CY, Nusse R (2004). The Wnt signaling pathway in development
and disease. Annu Rev Cell Dev Biol.

[5]  Cadigan KM, Peifer M (2009). Wnt signaling from development to disease:
insights from model systems. Cold Spring Harb Perspect Biol.

[6]  van Amerongen R, Nusse R (2009). Towards an integrated view of Wnt
signaling in development. Development.

[7]  MacDonald BT, Tamai K, He X (2009). Wnt/beta-catenin signaling: components,
mechanisms, and diseases. Dev Cell.

[8]  Clevers H (2006). Wnt/beta-catenin signaling in development and disease. Cell.

[9]  Polakis P (2007). The many ways of Wnt in cancer. Curr Opin Genet Dev.

[10]  Clevers H, Nusse R (2012). Wnt/β-catenin signaling and disease. Cell.

[11]  Polakis P (2012). Drugging Wnt signalling in cancer. EMBO J.

[12]  Behrens J, von Kries JP, Kuhl M, Bruhn L, Wedlich D, Grosschedl 
R (1996). Functional interaction of beta-catenin with the transcription
factor LEF-1. Nature.

[13]  Molenaar M, van de Wetering M, Oosterwegel  M, Peterson-Maduro  J, Godsave  S, Korinek V (1996). XTcf-3 transcription factor
mediates beta-catenin-induced axis formation in Xenopus embryos. Cell.

[14]  van de Wetering M, Sancho E, Verweij C, de LW, Oving I, Hurlstone 
A (2002). The beta-catenin/TCF-4 complex imposes a crypt
progenitor phenotype on colorectal cancer cells. Cell.

[15]  Xu W, Kimelman D (2007). Mechanistic insights from structural studies of
beta-catenin and its binding partners. J Cell Sci.

[16]  Coates JC (2003). Armadillo repeat proteins: beyond the animal kingdom. Trends Cell Biol.

[17]  Tewari R, Bailes E, Bunting KA, Coates JC (2010). Armadillo-repeat
protein functions: questions for little creatures. Trends Cell Biol.

[18]  Huber AH, Nelson WJ, Weis WI (1997). Three-dimensional structure of
the armadillo repeat region of beta-catenin. Cell.

[19]  Daugherty RL, Gottardi CJ (2007). Phospho-regulation of Beta-catenin
adhesion and signaling functions. Physiology (Bethesda).

[20]  Verheyen EM, Gottardi CJ (2010). Regulation of Wnt/beta-catenin signaling
by protein kinases. Dev Dyn.

[21]  Yost C, Torres M, Miller JR, Huang E, Kimelman D, Moon RT (1996). The axis-inducing activity, stability, and subcellular distribution of
beta-catenin is regulated in Xenopus embryos by glycogen synthase
kinase 3. Genes Dev.

[22]  Liu C, Li Y, Semenov M, Han C, Baeg GH, Tan Y (2002). Control of
beta-catenin phosphorylation/degradation by a dual-kinase mechanism. Cell.

[23]  Kitagawa M, Hatakeyama S, Shirane M, Matsumoto M, Ishida N, 
Hattori K (1999). An F-box protein, FWD1, mediates ubiquit-independent
proteolysis of beta-catenin. EMBO J.

[24]  Xing Y, Clements WK, Kimelman D, Xu W (2003). Crystal structure of a
beta-catenin/axin complex suggests a mechanism for the beta-catenin
destruction complex. Genes Dev.

[25]  Hart M, Concordet JP, Lassot I, Albert I, del los SR, Durand H (1999). The F-box protein beta-TrCP associates with phosphorylated
beta-catenin and regulates its activity in the cell. Curr Biol.

[26]  van Noort M, van de Wetering M, Clevers H (2002). Identification of two
novel regulated serines in the N terminus of beta-catenin. Exp Cell
Res.

[27]  van Noort M, Meeldijk J, van der Zee R, Destree O, Clevers H (2002). Wnt signaling controls the phosphorylation status of beta-catenin. J
Biol Chem.

[28]  Satoh S, Daigo Y, Furukawa Y, Kato T, Miwa N, Nishiwaki T (2000). AXIN1 mutations in hepatocellular carcinomas, and growth suppression
in cancer cells by virus-mediated transfer of AXIN1. Nat
Genet.

[29]  Webster MT, Rozycka M, Sara E, Davis E, Smalley M, Young N (2000). Sequence variants of the axin gene in breast, colon, and other
cancers: an analysis of mutations that interfere with GSK3 binding. Genes Chromosomes Cancer.

[30]  Dahmen RP, Koch A, Denkhaus D, Tonn JC, Sorensen N, Berthold 
F (2001). Deletions of AXIN1, a component of the WNT/wingless
pathway, in sporadic medulloblastomas. Cancer Res.

[31]  Clevers H (2000). Axin and hepatocellular carcinomas. Nat Genet.

[32]  Liu W, Dong X, Mai M, Seelan RS, Taniguchi K, 
Krishnadath KK (2000). Mutations in AXIN2 cause colorectal cancer with defective
mismatch repair by activating beta-catenin/TCF signalling. Nat Genet.

[33]  Wu R, Zhai Y, Fearon ER, Cho KR (2001). Diverse mechanisms of beta-catenin
deregulation in ovarian endometrioid adenocarcinomas. Cancer Res.

[34]  Parveen N, Hussain MU, Pandith AA, Mudassar S (2011). Diversity of
axin in signaling pathways and its relation to colorectal cancer. Med
Oncol.

[35]  Luo W, Lin SC (2004). Axin: a master scaffold for multiple signaling
pathways. Neurosignals.

[36]  Lee E, Salic A, Kruger R, Heinrich R, Kirschner MW (2003). The roles of
APC and Axin derived from experimental and theoretical analysis
of the Wnt pathway. PLoS Biol.

[37]  Huang SM, Mishina YM, Liu S, Cheung A, Stegmeier F, Michaud 
GA (2009). Tankyrase inhibition stabilizes axin and antagonizes Wnt
signalling. Nature.

[38]  Chen B, Dodge ME, Tang W, Lu J, Ma Z, Fan CW (2009). Small
molecule-mediated disruption of Wnt-dependent signaling in tissue
regeneration and cancer. Nat Chem Biol.

[39]  Waaler J, Machon O, von Kries JP, Wilson SR, Lundenes E, Wedlich 
D (2011). Novel synthetic antagonists of canonical Wnt signaling
inhibit colorectal cancer cell growth. Cancer Res.

[40]  Waaler J, Machon O, Tumova L, Dinh H, Korinek V, Wilson SR (2012). A novel tankyrase inhibitor decreases canonical Wnt signaling in
colon carcinoma cells and reduces tumor growth in conditional
APC mutant mice. Cancer Res.

[41]  Behrens J, Jerchow BA, Wurtele M, Grimm J, Asbrand C, Wirtz R (1998). Functional interaction of an axin homolog, conductin, with
beta-catenin, APC, and GSK3beta. Science.

[42]  von Kries JP, Winbeck G, Asbrand C, Schwarz-Romond T, 
Sochnikova N, Dell'Oro A (2000). Hot spots in beta-catenin for interactions
with LEF-1, conductin and APC. Nat Struct Biol.

[43]  Lustig B, Jerchow B, Sachs M, Weiler S, Pietsch T, Karsten U (2002). Negative feedback loop of Wnt signaling through upregulation of
conductin/axin2 in colorectal and liver tumors. Mol Cell Biol.

[44]  Leung JY, Kolligs FT, Wu R, Zhai Y, Kuick R, Hanash S (2002). Activation of AXIN2 expression by beta-catenin-T cell factor. A
feedback repressor pathway regulating Wnt signaling. J Biol Chem.

[45]  Hsu W, Zeng L, Costantini F (1999). Identification of a domain of Axin
that binds to the serine/threonine protein phosphatase 2A and a self-binding
domain. J Biol Chem.

[46]  Fiedler M, Mendoza-Topaz C, Rutherford TJ, Mieszczanek J, Bienz 
M (2011). Dishevelled interacts with the DIX domain polymerization interface
of Axin to interfere with its function in down-regulating beta-catenin. Proc Natl Acad Sci U S A.

[47]  Cliffe A, Hamada F, Bienz M (2003). A role of Dishevelled in relocating
Axin to the plasma membrane during wingless signaling. Curr Biol.

[48]  Schwarz-Romond T, Fiedler M, Shibata N, Butler PJ, Kikuchi A, 
Higuchi Y (2007). The DIX domain of Dishevelled confers Wnt signaling
by dynamic polymerization. Nat Struct Mol Biol.

[49]  Luo W, Zou H, Jin L, Lin S, Li Q, Ye Z (2005). Axin contains three
separable domains that confer intramolecular, homodimeric, and
heterodimeric interactions involved in distinct functions. J Biol
Chem.

[50]  Druey KM, Blumer KJ, Kang VH, Kehrl JH (1996). Inhibition of G-protein-
mediated MAP kinase activation by a new mammalian gene
family. Nature.

[51]  De Vries L, Zheng B, Fischer T, Elenko E, Farquhar MG (2000). The
regulator of G protein signaling family. Annu Rev Pharmacol Toxicol.

[52]  Castellone MD, Teramoto H, Williams BO, Druey KM, Gutkind JS (2005). Prostaglandin E2 promotes colon cancer cell growth through a Gsaxin-
beta-catenin signaling axis. Science.

[53]  Liu X, Rubin JS, Kimmel AR (2005). Rapid, Wnt-induced changes in
GSK3beta associations that regulate beta-catenin stabilization are
mediated by Galpha proteins. Curr Biol.

[54]  Egger-Adam D, Katanaev VL (2010). The trimeric G protein Go inflicts a
double impact on axin in the Wnt/frizzled signaling pathway. Dev Dyn.

[55]  Ikeda S, Kishida S, Yamamoto H, Murai H, Koyama S, Kikuchi A (1998). Axin, a negative regulator of the Wnt signaling pathway, forms a
complex with GSK-3beta and beta-catenin and promotes GSK-
3beta-dependent phosphorylation of beta-catenin. EMBO J.

[56]  Kodama S, Ikeda S, Asahara T, Kishida M, Kikuchi A (1999). Axin directly
interacts with plakoglobin and regulates its stability. J Biol
Chem.

[57]  Morrone S, Cheng Z, Moon RT, Cong F, Xu W (2012). Crystal structure of
a Tankyrase-Axin complex and its implications for Axin turnover
and Tankyrase substrate recruitment. Proc Natl Acad Sci U S A.

[58]  Yamamoto H, Kishida S, Kishida M, Ikeda S, Takada S, Kikuchi A (1999). Phosphorylation of axin, a Wnt signal negative regulator, by glycogen
synthase kinase-3beta regulates its stability. J Biol Chem.

[59]  Jho E, Lomvardas S, Costantini F (1999). A GSK3beta phosphorylation
site in axin modulates interaction with beta-catenin and Tcf-mediated
gene expression. Biochem Biophys Res Commun.

[60]  Luo W, Peterson A, Garcia BA, Coombs G, Kofahl B, Heinrich R (2007). Protein phosphatase 1 regulates assembly and function of the
beta-catenin degradation complex. EMBO J.

[61]  Willert K, Shibamoto S, Nusse R (1999). Wnt-induced dephosphorylation
of axin releases beta-catenin from the axin complex. Genes Dev.

[62]  Strovel ET, Wu D, Sussman DJ (2000). Protein phosphatase 2Calpha
dephosphorylates axin and activates LEF-1-dependent transcription. J Biol Chem.

[63]  Su Y, Fu C, Ishikawa S, Stella A, Kojima M, Shitoh K (2008). APC is
essential for targeting phosphorylated beta-catenin to the SCFbeta-
TrCP ubiquitin ligase. Mol Cell.

[64]  Ratcliffe MJ, Itoh K, Sokol SY (2000). A positive role for the PP2A catalytic
subunit in Wnt signal transduction. J Biol Chem.

[65]  Seeling JM, Miller JR, Gil R, Moon RT, White R, Virshup DM (1999). Regulation of beta-catenin signaling by the B56 subunit of protein
phosphatase 2A. Science.

[66]  Ikeda S, Kishida M, Matsuura Y, Usui H, Kikuchi A (2000). GSK-3beta-dependent
phosphorylation of adenomatous polyposis coli gene
product can be modulated by beta-catenin and protein phosphatase
2A complexed with Axin. Oncogene.

[67]  Zhang Y, Liu S, Mickanin C, Feng Y, Charlat O, Michaud GA (2011). RNF146 is a poly(ADP-ribose)-directed E3 ligase that regulates
axin degradation and Wnt signalling. Nat Cell Biol.

[68]  Callow MG, Tran H, Phu L, Lau T, Lee J, Sandoval WN (2011). Ubiquitin ligase RNF146 regulates tankyrase and Axin to promote
Wnt signaling. PLoS One.

[69]  Kim MJ, Chia IV, Costantini F (2008). SUMOylation target sites at the C
terminus protect Axin from ubiquitination and confer protein stability. FASEB J.

[70]  Kadoya T, Kishida S, Fukui A, Hinoi T, Michiue T, Asashima M (2000). Inhibition of Wnt signaling pathway by a novel axin-binding
protein. J Biol Chem.

[71]  Kadoya T, Yamamoto H, Suzuki T, Yukita A, Fukui A, Michiue T (2002). Desumoylation activity of Axam, a novel Axin-binding protein,
is involved in downregulation of beta-catenin. Mol Cell Biol.

[72]  Yamamoto H, Ihara M, Matsuura Y, Kikuchi A (2003). Sumoylation is
involved in beta-catenin-dependent activation of Tcf-4. EMBO J.

[73]  Cha B, Kim W, Kim YK, Hwang BN, Park SY, Yoon JW (2011). Methylation by protein arginine methyltransferase 1 increases stability
of Axin, a negative regulator of Wnt signaling. Oncogene.

[74]  Cong F, Varmus H (2004). Nuclear-cytoplasmic shuttling of Axin regulates
subcellular localization of beta-catenin. Proc Natl Acad Sci U S A.

[75]  Kaplan DD, Meigs TE, Kelly P, Casey PJ (2004). Identification of a role
for beta-catenin in the establishment of a bipolar mitotic spindle. J
Biol Chem.

[76]  Fodde R, Smits R, Clevers H (2001). APC, signal transduction and genetic
instability in colorectal cancer. Nat Rev Cancer.

[77]  Green RA, Wollman R, Kaplan KB (2005). APC and EB1 function together
in mitosis to regulate spindle dynamics and chromosome
alignment. Mol Biol Cell.

[78]  Wakefield JG, Stephens DJ, Tavare JM (2003). A role for glycogen synthase
kinase-3 in mitotic spindle dynamics and chromosome alignment. J Cell Sci.

[79]  Kim SM, Choi EJ, Song KJ, Kim S, Seo E, Jho EH (2009). Axin
localizes to mitotic spindles and centrosomes in mitotic cells. Exp
Cell Res.

[80]  Guo X, Ramirez A, Waddell DS, Li Z, Liu X, Wang XF (2008). Axin and
GSK3-β control Smad3 protein stability and modulate TGF-beta
signaling. Genes Dev.

[81]  Zhang M, Wang M, Tan X, Li TF, Zhang YE, Chen D (2010). Smad3
prevents beta-catenin degradation and facilitates beta-catenin nuclear
translocation in chondrocytes. J Biol Chem.

[82]  Santoro IM, Groden J (1997). Alternative splicing of the APC gene and its
association with terminal differentiation. Cancer Res.

[83]  Fodde R, Edelmann W, Yang K, van Leeuwen C, Carlson C Renault
B (1994). A targeted chain-termination mutation in the mouse
Apc gene results in multiple intestinal tumors. Proc Natl Acad Sci
U S A.

[84]  Tejpar S, Michils G, Denys H, Van Dam K, Nik SA, Jadidizadeh A (2005). Analysis of Wnt/Beta catenin signalling in desmoid tumors. Acta Gastroenterol Belg.

[85]  Polakis P (1997). The adenomatous polyposis coli (APC) tumor suppressor. Biochim Biophys Acta.

[86]  Polakis P (2000). Wnt signaling and cancer. Genes Dev.

[87]  Rubinfeld B, Tice DA, Polakis P (2001). Axin-dependent phosphorylation
of the adenomatous polyposis coli protein mediated by casein
kinase 1epsilon. J Biol Chem.

[88]  Ha NC, Tonozuka T, Stamos JL, Choi HJ, Weis WI (2004). Mechanism of
phosphorylation-dependent binding of APC to beta-catenin and its
role in beta-catenin degradation. Mol Cell.

[89]  Henderson BR (2000). Nuclear-cytoplasmic shuttling of APC regulates
beta-catenin subcellular localization and turnover. Nat Cell Biol.

[90]  Rosin-Arbesfeld R, Townsley F, Bienz M (2000). The APC tumour suppressor
has a nuclear export function. Nature.

[91]  Neufeld KL, Zhang F, Cullen BR, White RL (2000). APC-mediated down-regulation
of beta-catenin activity involves nuclear sequestration
and nuclear export. EMBO Rep.

[92]  Zhang F, White RL, Neufeld KL (2000). Phosphorylation near nuclear
localization signal regulates nuclear import of adenomatous polyposis
coli protein. Proc Natl Acad Sci U S A.

[93]  Galea MA, Eleftheriou A, Henderson BR (2001). ARM domain-dependent
nuclear import of adenomatous polyposis coli protein is stimulated
by the B56 alpha subunit of protein phosphatase 2A. J Biol Chem.

[94]  Neufeld KL (2009). Nuclear APC. Adv Exp Med Biol.

[95]  Hulsken J, Birchmeier W, Behrens J (1994). E-cadherin and APC compete
for the interaction with beta-catenin and the cytoskeleton. J Cell
Biol.

[96]  Munemitsu S, Souza B, Muller O, Albert I, Rubinfeld B, Polakis P (1994). The APC gene product associates with microtubules *in vivo* and
promotes their assembly *in vitro*. Cancer Res.

[97]  Su LK, Burrell M, Hill DE, Gyuris J, Brent R, Wiltshire R (1995). APC binds to the novel protein EB1. Cancer Res.

[98]  Matsumine A, Ogai A, Senda T, Okumura N, Satoh K, Baeg GH (1996). Binding of APC to the human homolog of the Drosophila discs
large tumor suppressor protein. Science.

[99]  Embi N, Rylatt DB, Cohen P (1980). Glycogen synthase kinase-3 from
rabbit skeletal muscle. Separation from cyclic-AMP-dependent protein
kinase and phosphorylase kinase. Eur J Biochem.

[100]  Mukai F, Ishiguro K, Sano Y, Fujita SC (2002). Alternative splicing isoform
of tau protein kinase I/glycogen synthase kinase 3beta. J
Neurochem.

[101]  Desbois-Mouthon C, Blivet-Van Eggelpoel MJ, Beurel E, Boissan 
M, Delelo R, Cadoret A (2002). Dysregulation of glycogen synthase
kinase-3beta signaling in hepatocellular carcinoma cells. Hepatology.

[102]  Goto H, Kawano K, Kobayashi I, Sakai H, Yanagisawa S (2002). Expression
of cyclin D1 and GSK-3beta and their predictive value of
prognosis in squamous cell carcinomas of the tongue. Oral Oncol.

[103]  Mulholland DJ, Dedhar S, Wu H, Nelson CC (2006). PTEN and
GSK3beta: key regulators of progression to androgen-independent
prostate cancer. Oncogene.

[104]  Osolodkin DI, Palyulin VA, Zefirov NS (2013). Glycogen synthase kinase
3 as an anticancer drug target: Novel experimental findings and
trends in the design of inhibitors. Curr Pharm Des.

[105]  Jope RS, Roh MS (2006). Glycogen synthase kinase-3 (GSK3) in psychiatric
diseases and therapeutic interventions. Curr Drug Targets.

[106]  Jope RS, Yuskaitis CJ, Beurel E (2007). Glycogen synthase kinase-3
(GSK3): inflammation, diseases, and therapeutics. Neurochem Res.

[107]  Rayasam GV, Tulasi VK, Sodhi R, Davis JA, Ray A (2009). Glycogen
synthase kinase 3: more than a namesake. Br J Pharmacol.

[108]  Hoeflich KP, Luo J, Rubie EA, Tsao MS, Jin O, Woodgett JR (2000). Requirement for glycogen synthase kinase-3beta in cell survival
and NF-kappaB activation. Nature.

[109]  Sutherland C (2011). What Are the bona fide GSK3 Substrates?. Int J Alzheimers Dis.

[110]  Blair K, Wray J, Smith A (2011). The liberation of embryonic stem cells. PLoS Genet.

[111]  Kim WY, Wang X, Wu Y, Doble BW, Patel S, Woodgett JR (2009). GSK-3 is a master regulator of neural progenitor homeostasis. Nat
Neurosci.

[112]  Doble BW, Patel S, Wood GA, Kockeritz LK, Woodgett JR (2007). Functional
redundancy of GSK-3alpha and GSK-3beta in Wnt/beta-catenin
signaling shown by using an allelic series of embryonic
stem cell lines. Dev Cell.

[113]  Wada A (2009). GSK-3 inhibitors and insulin receptor signaling in health,
disease, and therapeutics. Front Biosci.

[114]  Force T, Woodgett JR (2009). Unique and overlapping functions of GSK-3
isoforms in cell differentiation and proliferation and cardiovascular
development. J Biol Chem.

[115]  Dominguez I, Itoh K, Sokol SY (1995). Role of glycogen synthase kinase 3
beta as a negative regulator of dorsoventral axis formation in
Xenopus embryos. Proc Natl Acad Sci U S A.

[116]  Jiang J, Struhl G (1998). Regulation of the Hedgehog and Wingless signalling
pathways by the F-box/WD40-repeat protein Slimb. Nature.

[117]  Marikawa Y, Elinson RP (1998). beta-TrCP is a negative regulator of
Wnt/beta-catenin signaling pathway and dorsal axis formation in
Xenopus embryos. Mech Dev.

[118]  Latres E, Chiaur DS, Pagano M (1999). The human F box protein beta-
Trcp associates with the Cul1/Skp1 complex and regulates the stability
of beta-catenin. Oncogene.

[119]  Sadot E, Simcha I, Iwai K, Ciechanover A, Geiger B, Ben-Ze'ev A (2000). Differential interaction of plakoglobin and beta-catenin with the
ubiquitin-proteasome system. Oncogene.

[120]  Teuliere J, Faraldo MM, Shtutman M, Birchmeier W, Huelsken J, Thiery JP (2004). beta-catenin-dependent and -independent effects of
DeltaN-plakoglobin on epidermal growth and differentiation. Mol
Cell Biol.

[121]  ter Haar E, Coll JT, Austen DA, Hsiao HM, Swenson L, Jain J (2001). Structure of GSK3beta reveals a primed phosphorylation mechanism. Nat Struct Biol.

[122]  Thornton TM, Pedraza-Alva G, Deng B, Wood CD, Aronshtam A, 
Clements JL (2008). Phosphorylation by p38 MAPK as an alternative
pathway for GSK3beta inactivation. Science.

[123]  Cole A, Frame S, Cohen P (2004). Further evidence that the tyrosine phosphorylation
of glycogen synthase kinase-3 (GSK3) in mammalian
cells is an autophosphorylation event. Biochem J.

[124]  Yost C, Farr GH, Pierce SB, Ferkey DM, Chen MM, Kimelman D (1998). GBP, an inhibitor of GSK-3, is implicated in Xenopus development
and oncogenesis. Cell.

[125]  Li L, Yuan H, Weaver CD, Mao J, Farr GH, Sussman DJ (1999). Axin and Frat1 interact with dvl and GSK, bridging Dvl to GSK in
Wnt-mediated regulation of LEF-1. EMBO J.

[126]  Freemantle SJ, Portland HB, Ewings K, Dmitrovsky F, DiPetrillo 
K, Spinella MJ (2002). Characterization and tissue-specific expression
of human GSK-3-binding proteins FRAT1 and FRAT2. Gene.

[127]  Wang Y, Liu S, Zhu H, Zhang W, Zhang G, Zhou X (2008). FRAT1
overexpression leads to aberrant activation of beta-catenin/TCF
pathway in esophageal squamous cell carcinoma. Int J Cancer.

[128]  Guo G, Liu B, Zhong C, Zhang X, Mao X, Wang P (2011). FRAT1
expression and its correlation with pathologic grade, proliferation,
and apoptosis in human astrocytomas. Med Oncol.

[129]  Guo G, Mao X, Wang P, Liu B, Zhang X, Jiang X (2010). The expression
profile of FRAT1 in human gliomas. Brain Res.

[130]  Zhang Y, Yu JH, Lin XY, Miao Y, Han Y, Fan CF (2011). Overexpression
of Frat1 correlates with malignant phenotype and advanced
stage in human non-small cell lung cancer. Virchows Arch.

[131]  van Amerongen R, Nawijn MC, Lambooij JP, Proost N, Jonkers J, 
Berns A (2010). Frat oncoproteins act at the crossroad of canonical and
noncanonical Wnt-signaling pathways. Oncogene.

[132]  Flugel D, Gorlach A, Michiels C, Kietzmann T (2007). Glycogen synthase
kinase 3 phosphorylates hypoxia-inducible factor 1alpha and mediates
its destabilization in a VHL-independent manner. Mol Cell
Biol.

[133]  Zhou BP, Deng J, Xia W, Xu J, Li YM, Gunduz M (2004). Dual
regulation of Snail by GSK-3beta-mediated phosphorylation in control
of epithelial-mesenchymal transition. Nat Cell Biol.

[134]  Hernandez F, Nido JD, Avila J, Villanueva N (2009). GSK3 inhibitors and
disease. Mini Rev Med Chem.

[135]  Garcia I, Fall Y, Gomez G (2010). QSAR, docking, and CoMFA studies of
GSK3 inhibitors. Curr Pharm Des.

[136]  Plotkin B, Kaidanovich O, Talior I, Eldar-Finkelman H (2003). Insulin
mimetic action of synthetic phosphorylated peptide inhibitors of
glycogen synthase kinase-3. J Pharmacol Exp Ther.

[137]  Bikkavilli RK, Feigin ME, Malbon CC (2008). p38 mitogen-activated
protein kinase regulates canonical Wnt-beta-catenin signaling by
inactivation of GSK3beta. J Cell Sci.

[138]  Anand P, Shenoy R, Palmer JE, Baines AJ, Lai RY, Robertson J (2011). Clinical trial of the p38 MAP kinase inhibitor dilmapimod in
neuropathic pain following nerve injury. Eur J Pain.

[139]  Xing L, Devadas B, Devraj RV, Selness SR, Shieh H, Walker JK (2012). Discovery and characterization of atropisomer PH-797804, a
p38 MAP kinase inhibitor, as a clinical drug candidate. ChemMedChem.

[140]  Smith S, Giriat I, Schmitt A, de Lange T (1998). Tankyrase, a poly(ADPribose)
polymerase at human telomeres. Science.

[141]  Hsiao SJ, Smith S (2008). Tankyrase function at telomeres, spindle poles,
and beyond. Biochimie.

[142]  Morrone S, Cheng Z, Moon RT, Cong F, Xu W (2012). Crystal structure of
a Tankyrase-Axin complex and its implications for Axin turnover
and Tankyrase substrate recruitment. Proc Natl Acad Sci U S A.

[143]  Kim CA, Phillips ML, Kim W, Gingery M, Tran H, Robinson MA (2001). Polymerization of the SAM domain of TEL in leukemogenesis
and transcriptional repression. EMBO J.

[144]  Kim CA, Gingery M, Pilpa RM, Bowie JU (2002). The SAM domain of
polyhomeotic forms a helical polymer. Nat Struct Biol.

[145]  De Rycker M, Price CM (2004). Tankyrase polymerization is controlled
by its sterile alpha motif and poly(ADP-ribose) polymerase domains. Mol Cell Biol.

[146]  Guettler S, LaRose J, Petsalaki E, Gish G, Scotter A, Pawson T (2011). Structural basis and sequence rules for substrate recognition by
Tankyrase explain the basis for cherubism disease. Cell.

[147]  Slade D, Dunstan MS, Barkauskaite E, Weston R, Lafite P, Dixon 
N (2011). The structure and catalytic mechanism of a poly(ADP-ribose)
glycohydrolase. Nature.

[148]  Okita N, Ashizawa D, Ohta R, Abe H, Tanuma S (2010). Discovery of
novel poly(ADP-ribose) glycohydrolase inhibitors by a quantitative
assay system using dot-blot with anti-poly(ADP-ribose). Biochem
Biophys Res Commun.

[149]  Dregalla RC, Zhou J, Idate RR, Battaglia CL, Liber HL, Bailey SM (2010). Regulatory roles of tankyrase 1 at telomeres and in DNA repair:
suppression of T-SCE and stabilization of DNA-PKcs. Aging (Albany NY).

[150]  Wang Z, Michaud GA, Cheng Z, Zhang Y, Hinds TR, Fan E (2012). Recognition of the iso-ADP-ribose moiety in poly(ADP-ribose) by
WWE domains suggests a general mechanism for poly(ADP-ribosyl)
ation-dependent ubiquitination. Genes Dev.

[151]  Ha GH, Kim HS, Go H, Lee H, Seimiya H, Chung DH (2012). Tankyrase-
1 function at telomeres and during mitosis is regulated by
Polo-like kinase-1-mediated phosphorylation. Cell Death Differ.

[152]  Kikuchi K, Niikura Y, Kitagawa K, Kikuchi A (2010). Dishevelled, a Wnt
signalling component, is involved in mitotic progression in cooperation
with Plk1. EMBO J.

[153]  Chi NW, Lodish HF (2000). Tankyrase is a golgi-associated mitogen-activated
protein kinase substrate that interacts with IRAP in
GLUT4 vesicles. J Biol Chem.

[154]  Waters SB, D'Auria M, Martin SS, Nguyen C, Kozma LM, Luskey 
KL (1997). The amino terminus of insulin-responsive aminopeptidase
causes Glut4 translocation in 3T3-L1 adipocytes. J Biol Chem.

[155]  Sbodio JI, Chi NW (2002). Identification of a tankyrase-binding motif
shared by IRAP, TAB182, and human TRF1 but not mouse TRF1.
NuMA contains this RXXPDG motif and is a novel tankyrase partner. J Biol Chem.

[156]  Radulescu S, Ridgway RA, Appleton P, Kroboth K, Patel S, 
Woodgett J (2010). Defining the role of APC in the mitotic spindle
checkpoint *in vivo*: APC-deficient cells are resistant to Taxol. Oncogene.

[157]  Yeh TY, Meyer TN, Schwesinger C, Tsun ZY, Lee RM, Chi NW (2006). Tankyrase recruitment to the lateral membrane in polarized epithelial
cells: regulation by cell-cell contact and protein poly(ADP-ribosyl)
ation. Biochem J.

[158]  Park JI, Venteicher AS, Hong JY, Choi J, Jun S, Shkreli M (2009). Telomerase modulates Wnt signalling by association with target
gene chromatin. Nature.

[159]  Gat U, DasGupta R, Degenstein L, Fuchs E (1998). De Novo hair follicle
morphogenesis and hair tumors in mice expressing a truncated beta-catenin
in skin. Cell.

[160]  Van Mater D, Kolligs FT, Dlugosz AA, Fearon ER (2003). Transient
activation of beta -catenin signaling in cutaneous keratinocytes is
sufficient to trigger the active growth phase of the hair cycle in
mice. Genes Dev.

[161]  Lo Celso C, Prowse DM, Watt FM (2004). Transient activation of beta-catenin
signalling in adult mouse epidermis is sufficient to induce
new hair follicles but continuous activation is required to maintain
hair follicle tumours. Development.

[162]  Millar SE (2009). Cell biology: The not-so-odd couple. Nature.

[163]  Ulsamer A, Wei Y, Kim KK, Tan K, Wheeler S, Xi Y (2012). Axin
pathway activity regulates *in vivo* pY654-beta-catenin accumulation
and pulmonary fibrosis. J Biol Chem.

[164]  Seto ES, Bellen HJ (2006). Internalization is required for proper Wingless
signaling in Drosophila melanogaster. J Cell Biol.

[165]  Karlberg T, Markova N, Johansson I, Hammarstrom M, Schutz P, 
Weigelt J (2010). Structural basis for the interaction between tankyrase-
2 and a potent Wnt-signaling inhibitor. J Med Chem.

[166]  Wahlberg E, Karlberg T, Kouznetsova E, Markova N, Macchiarulo 
A, Thorsell AG (2012). Family-wide chemical profiling and structural
analysis of PARP and tankyrase inhibitors. Nat Biotechnol.

[167]  Narwal M, Fallarero A, Vuorela P, Lehtio L (2012). Homogeneous screening
assay for human tankyrase. J Biomol Screen.

[168]  Shultz MD, Kirby CA, Stams T, Chin DN, Blank J, Charlat O (2012). [1,2,4]triazol-3-ylsulfanylmethyl)-3-phenyl-[1,2,4]oxadiazoles: antagonists
of the Wnt pathway that inhibit tankyrases 1 and 2 via
novel adenosine pocket binding. J Med Chem.

[169]  Gunaydin H, Gu Y, Huang X (2012). Novel binding mode of a potent and
selective tankyrase inhibitor. PLoS One.

[170]  Kirby CA, Cheung A, Fazal A, Shultz MD, Stams T (2012). Structure of
human tankyrase 1 in complex with small-molecule inhibitors PJ34
and XAV939. Acta Crystallogr Sect F Struct Biol Cryst Commun.

[171]  Dani N, Barbosa AJ, Del RA, Di GM (2012). ADP-Ribosylated Proteins as
Old and New Drug Targets for Anticancer Therapy: the Example of
ARF6. Curr Pharm Des.

[172]  Komekado H, Yamamoto H, Chiba T, Kikuchi A (2007). Glycosylation
and palmitoylation of Wnt-3a are coupled to produce an active form
of Wnt-3a. Genes Cells.

[173]  Smolich BD, McMahon JA, McMahon AP, Papkoff J (1993). Wnt family
proteins are secreted and associated with the cell surface. Mol Biol
Cell.

[174]  Yan Q, Lennarz WJ (1999). Oligosaccharyltransferase: a complex multisubunit
enzyme of the endoplasmic reticulum. Biochem Biophys
Res Commun.

[175]  Willert K, Brown JD, Danenberg E, Duncan AW, Weissman IL, 
Reya T (2003). Wnt proteins are lipid-modified and can act as stem
cell growth factors. Nature.

[176]  Buechling T, Boutros M (2011). Wnt signaling signaling at and above the
receptor level. Curr Top Dev Biol.

[177]  Takada R, Satomi Y, Kurata T, Ueno N, Norioka S, Kondoh H (2006). Monounsaturated fatty acid modification of Wnt protein: its role
in Wnt secretion. Dev Cell.

[178]  Mason JO, Kitajewski J, Varmus HE (1992). Mutational analysis of mouse
Wnt-1 identifies two temperature-sensitive alleles and attributes of
Wnt-1 protein essential for transformation of a mammary cell line. Mol Biol Cell.

[179]  Franch-Marro X, Wendler F, Griffith J, Maurice MM, Vincent JP (2008). *In vivo* role of lipid adducts on Wingless. J Cell Sci.

[180]  Port F, Basler K (2010). Wnt trafficking: new insights into Wnt maturation,
secretion and spreading. Traffic.

[181]  Banziger C, Soldini D, Schutt C, Zipperlen P, Hausmann G, Basler 
K (2006). Wntless, a conserved membrane protein dedicated to the secretion
of Wnt proteins from signaling cells. Cell.

[182]  Bartscherer K, Pelte N, Ingelfinger D, Boutros M (2006). Secretion of Wnt
ligands requires Evi, a conserved transmembrane protein. Cell.

[183]  Goodman RM, Thombre S, Firtina Z, Gray D, Betts D (2006). Roebuck Sprinter: a novel transmembrane protein required for Wg secretion
and signaling. Development.

[184]  Adell T, Salo E, Boutros M, Bartscherer K (2009). Smed-Evi/Wntless is
required for beta-catenin-dependent and -independent processes
during planarian regeneration. Development.

[185]  Coombs GS, Yu J, Canning CA, Veltri CA, Covey TM, Cheong JK (2010). WLS-dependent secretion of WNT3A requires Ser209 acylation
and vacuolar acidification. J Cell Sci.

[186]  Fu J, Jiang M, Mirando AJ, Yu HM, Hsu W (2009). Reciprocal regulation
of Wnt and Gpr177/mouse Wntless is required for embryonic axis
formation. Proc Natl Acad Sci U S A.

[187]  Yu HM, Jin Y, Fu J, Hsu W (2010). Expression of Gpr177, a Wnt trafficking
regulator, in mouse embryogenesis. Dev Dyn.

[188]  Carpenter AC, Rao S, Wells JM, Campbell K, Lang RA (2010). Generation
of mice with a conditional null allele for Wntless. Genesis.

[189]  Belenkaya TY, Wu Y, Tang X, Zhou B, Cheng L, Sharma YV (2008). The retromer complex influences Wnt secretion by recycling
wntless from endosomes to the trans-Golgi network. Dev Cell.

[190]  Niehrs C, Boutros M (2010). Trafficking, acidification, and growth factor
signaling. Sci Signal.

[191]  Tanaka K, Kitagawa Y, Kadowaki T (2002). Drosophila segment polarity
gene product porcupine stimulates the posttranslational N-glycosylation
of wingless in the endoplasmic reticulum. J Biol
Chem.

[192]  Zhai L, Chaturvedi D, Cumberledge S (2004). Drosophila wnt-1 undergoes
a hydrophobic modification and is targeted to lipid rafts, a process
that requires porcupine. J Biol Chem.

[193]  Zoltewicz JS, Ashique AM, Choe Y, Lee G, Taylor S, Phamluong 
K (2009). Wnt signaling is regulated by endoplasmic reticulum retention. PLoS One.

[194]  Dodge ME, Moon J, Tuladhar R, Lu J, Jacob LS, Zhang LS (2012). Diverse chemical scaffolds support direct inhibition of the membrane
bound O-acyltransferase Porcupine. J Biol Chem.

[195]  Zecca M, Basler K, Struhl G (1996). Direct and long-range action of a
wingless morphogen gradient. Cell.

[196]  Fuerer C, Habib SJ, Nusse R (2010). A study on the interactions between
heparan sulfate proteoglycans and Wnt proteins. Dev Dyn.

[197]  Panakova D, Sprong H, Marois E, Thiele C, Eaton S (2005). Lipoprotein
particles are required for Hedgehog and Wingless signalling. Nature.

[198]  Korkut C, Ataman B, Ramachandran P, Ashley J, Barria R, Gherbesi 
N (2009). Trans-synaptic transmission of vesicular Wnt signals
through Evi/Wntless. Cell.

[199]  Yan D, Lin X (2009). Shaping morphogen gradients by proteoglycans. Cold Spring Harb Perspect Biol.

[200]  Greco V, Hannus M, Eaton S (2001). Argosomes: a potential vehicle for
the spread of morphogens through epithelia. Cell.

[201]  Rao TP, Kuhl M (2010). An updated overview on Wnt signaling pathways:
a prelude for more. Circ Res.

[202]  Tu X, Joeng KS, Nakayama KI, Nakayama K, Rajagopal J, Carroll 
TJ (2007). Noncanonical Wnt signaling through G protein-linked
PKCdelta activation promotes bone formation. Dev Cell.

[203]  He X, Saint-Jeannet JP, Wang Y, Nathans J, Dawid I, Varmus H (1997). A
member of the Frizzled protein family mediating axis induction by
Wnt-5A. Science.

[204]  Tao Q, Yokota C, Puck H, Kofron M, Birsoy B, Yan D (2005). Maternal
wnt11 activates the canonical wnt signaling pathway required
for axis formation in Xenopus embryos. Cell.

[205]  He X, Semenov M, Tamai K, Zeng X (2004). LDL receptor-related proteins
5 and 6 in Wnt/beta-catenin signaling: arrows point the way. Development.

[206]  Li Y, Bu G (2005). LRP5/6 in Wnt signaling and tumorigenesis. Future Oncol.

[207]  Nusse R, Varmus H (2012). Three decades of Wnts: a personal perspective
on how a scientific field developed. EMBO J.

[208]  Vinson CR, Conover S, Adler PN (1989). A Drosophila tissue polarity
locus encodes a protein containing seven potential transmembrane
domains. Nature.

[209]  Bhanot P, Brink M, Samos CH, Hsieh JC, Wang Y, Macke JP (1996). A new member of the frizzled family from Drosophila functions as
a Wingless receptor. Nature.

[210]  Tolwinski NS, Wehrli M, Rives A, Erdeniz N, DiNardo S, Wieschaus 
E (2003). Wg/Wnt signal can be transmitted through arrow/
LRP5,6 and Axin independently of Zw3/Gsk3beta activity. Dev Cell.

[211]  Cong F, Schweizer L, Varmus H (2004). Wnt signals across the plasma
membrane to activate the beta-catenin pathway by forming oligomers
containing its receptors, Frizzled and LRP. Development.

[212]  Holmen SL, Robertson SA, Zylstra CR, Williams BO (2005). Wnt-independent
activation of beta-catenin mediated by a Dkk1-Fz5 fusion
protein. Biochem Biophys Res Commun.

[213]  Caneparo L, Huang YL, Staudt N, Tada M, Ahrendt R, Kazanskaya 
O (2007). Dickkopf-1 regulates gastrulation movements by coordinated
modulation of Wnt/beta catenin and Wnt/PCP activities,
through interaction with the Dally-like homolog Knypek. Genes Dev.

[214]  Bryja V, Andersson ER, Schambony A, Esner M, Bryjova L, Biris 
KK (2009). The extracellular domain of Lrp5/6 inhibits noncanonical
Wnt signaling *in vivo*. Mol Biol Cell.

[215]  Tamai K, Semenov M, Kato Y, Spokony R, Liu C, Katsuyama Y (2000). LDL-receptor-related proteins in Wnt signal transduction. Nature.

[216]  Cheyette BN, Waxman JS, Miller JR, Takemaru K, Sheldahl LC, 
Khlebtsova N (2002). Dapper, a Dishevelled-associated antagonist of
beta-catenin and JNK signaling, is required for notochord formation. Dev Cell.

[217]  Bilic J, Huang YL, Davidson G, Zimmermann T, Cruciat CM, 
Bienz M (2007). Wnt induces LRP6 signalosomes and promotes
dishevelled-dependent LRP6 phosphorylation. Science.

[218]  Mao B, Wu W, Li Y, Hoppe D, Stannek P, Glinka A (2001). LDL-receptor-
related protein 6 is a receptor for Dickkopf proteins. Nature.

[219]  Davidson G, Wu W, Shen J, Bilic J, Fenger U, Stannek P (2005). Casein kinase 1 gamma couples Wnt receptor activation to cytoplasmic
signal transduction. Nature.

[220]  Tamai K, Zeng X, Liu C, Zhang X, Harada Y, Chang Z (2004). A
mechanism for Wnt coreceptor activation. Mol Cell.

[221]  Zeng X, Tamai K, Doble B, Li S, Huang H, Habas R (2005). A dual-kinase
mechanism for Wnt co-receptor phosphorylation and activation. Nature.

[222]  Wu H, Symes K, Seldin DC, Dominguez I (2009). Threonine 393 of beta-catenin
regulates interaction with Axin. J Cell Biochem.

[223]  Pan W, Choi SC, Wang H, Qin Y, Volpicelli-Daley L, Swan L (2008). Wnt3a-mediated formation of phosphatidylinositol 4,5-
bisphosphate regulates LRP6 phosphorylation. Science.

[224]  Katanaev VL, Ponzielli R, Semeriva M, Tomlinson A (2005). Trimeric G
protein-dependent frizzled signaling in Drosophila. Cell.

[225]  Katanaev VL, Tomlinson A (2006). Dual roles for the trimeric G protein
Go in asymmetric cell division in Drosophila. Proc Natl Acad Sci U S A.

[226]  Katanaev VL, Tomlinson A (2006). Multiple roles of a trimeric G protein
in Drosophila cell polarization. Cell Cycle.

[227]  Egger-Adam D, Katanaev VL (2008). Trimeric G protein-dependent signaling
by Frizzled receptors in animal development. Front Biosci.

[228]  Katanaev VL (2010). The Wnt/Frizzled GPCR signaling pathway. Biochemistry (Mosc ).

[229]  Purvanov V, Koval A, Katanaev VL (2010). A direct and functional interaction
between Go and Rab5 during G protein-coupled receptor
signaling. Sci Signal.

[230]  Del Valle-Perez B, Arques O, Vinyoles M, de Herreros AG, Dunach M (2011). Coordinated action of CK1 isoforms in canonical Wnt
signaling. Mol Cell Biol.

[231]  Cselenyi CS, Jernigan KK, Tahinci E, Thorne CA, Lee LA, Lee E (2008). LRP6 transduces a canonical Wnt signal independently of Axin
degradation by inhibiting GSK3's phosphorylation of beta-catenin. Proc Natl Acad Sci U S A.

[232]  Cong F, Schweizer L, Varmus H (2004). Casein kinase Iepsilon modulates
the signaling specificities of dishevelled. Mol Cell Biol.

[233]  Zhai L, Graves PR, Robinson LC, Italiano M, Culbertson MR, Rowles J (1995). Casein kinase I gamma subfamily. Molecular cloning,
expression, and characterization of three mammalian isoforms
and complementation of defects in the Saccharomyces cerevisiae
YCK genes. J Biol Chem.

[234]  Swiatek W, Tsai IC, Klimowski L, Pepler A, Barnette J, Yost HJ (2004). Regulation of casein kinase I epsilon activity by Wnt signaling. J
Biol Chem.

[235]  Liang J, Fu Y, Cruciat CM, Jia S, Wang Y, Tong Z (2011). Transmembrane
protein 198 promotes LRP6 phosphorylation and Wnt
signaling activation. Mol Cell Biol.

[236]  Niehrs C, Shen J (2010). Regulation of Lrp6 phosphorylation. Cell Mol Life Sci.

[237]  Cervenka I, Wolf J, Masek J, Krejci P, Wilcox WR, Kozubik A (2011). Mitogen-activated protein kinases promote WNT/beta-catenin
signaling via phosphorylation of LRP6. Mol Cell Biol.

[238]  Krejci P, Aklian A, Kaucka M, Sevcikova E, Prochazkova J, Masek JK (2012). Receptor tyrosine kinases activate canonical WNT/beta-catenin
signaling via MAP kinase/LRP6 pathway and direct beta-catenin
phosphorylation. PLoS One.

[239]  Piao S, Lee SH, Kim H, Yum S, Stamos JL, Xu Y (2008). Direct
inhibition of GSK3beta by the phosphorylated cytoplasmic domain
of LRP6 in Wnt/beta-catenin signaling. PLoS One.

[240]  Kofron M, Birsoy B, Houston D, Tao Q, Wylie C, Heasman J (2007). Wnt11/beta-catenin signaling in both oocytes and early embryos
acts through LRP6-mediated regulation of axin. Development.

[241]  Taelman VF, Dobrowolski R, Plouhinec JL, Fuentealba LC, Vorwald PP, Gumper I (2010). Wnt signaling requires sequestration of
glycogen synthase kinase 3 inside multivesicular endosomes. Cell.

[242]  Blitzer JT, Nusse R (2006). A critical role for endocytosis in Wnt signaling. BMC Cell Biol.

[243]  Yamamoto H, Komekado H, Kikuchi A (2006). Caveolin is necessary for
Wnt-3a-dependent internalization of LRP6 and accumulation of
beta-catenin. Dev Cell.

[244]  de Lau W, Barker N, Low TY, Koo BK, Li VS, Teunissen H (2011). Lgr5 homologues associate with Wnt receptors and mediate R-spondin
signalling. Nature.

[245]  Carmon KS, Gong X, Lin Q, Thomas A, Liu Q (2011). R-spondins function
as ligands of the orphan receptors LGR4 and LGR5 to regulate
Wnt/beta-catenin signaling. Proc Natl Acad Sci U S A.

[246]  Glinka A, Dolde C, Kirsch N, Huang YL, Kazanskaya O, Ingelfinger D (2011). LGR4 and LGR5 are R-spondin receptors mediating
Wnt/beta-catenin and Wnt/PCP signalling. EMBO Rep.

[247]  Romero G, Sneddon WB, Yang Y, Wheeler D, Blair HC, Friedman 
PA (2010). Parathyroid hormone receptor directly interacts with dishevelled
to regulate beta-Catenin signaling and osteoclastogenesis. J Biol Chem.

[248]  Gensure RC, Gardella TJ, Juppner H (2005). Parathyroid hormone and
parathyroid hormone-related peptide, and their receptors. Biochem
Biophys Res Commun.

[249]  Barker N, Huch M, Kujala P, van de Wetering M, Snippert HJ, van Es JH (2010). Lgr5(+ve) stem cells drive self-renewal in the
stomach and build long-lived gastric units *in vitro*. Cell Stem Cell.

[250]  He B, Jablons DM (2006). Wnt signaling in stem cells and lung cancer. Ernst Schering Found Symp Proc.

[251] Gurney A, Oncomed Pharmaceuticals Inc.; 2007 (2012). METHODS FOR
DIAGNOSING AND TREATING CANCER(FR) COMPOSITIONS
ET PROCÉDÉS DE DIAGNOSTIC ET DE TRAITEMENT
DU CANCER. Patent WO2007142711.

[252]  Nagayama S, Fukukawa C, Katagiri T, Okamoto T, Aoyama T, Oyaizu N (2005). Therapeutic potential of antibodies against FZD 10,
a cell-surface protein, for synovial sarcomas. Oncogene.

[253]  Chen M, Wang J, Lu J, Bond MC, Ren XR, Lyerly HK (2009). The
anti-helminthic niclosamide inhibits Wnt/Frizzled1 signaling. Biochemistry.

[254]  Lu W, Lin C, Roberts MJ, Waud WR, Piazza GA, Li Y (2011). Niclosamide
suppresses cancer cell growth by inducing Wnt co-receptor
LRP6 degradation and inhibiting the Wnt/beta-catenin
pathway. PLoS One.

[255]  Pan JX, Ding K, Wang CY (2012). Niclosamide, an old antihelminthic
agent, demonstrates antitumor activity by blocking multiple signaling
pathways of cancer stem cells. Chin J Cancer.

[256]  Osada T, Chen M, Yang XY, Spasojevic I, Vandeusen JB, Hsu D (2011). Antihelminth compound niclosamide downregulates Wnt signaling
and elicits antitumor responses in tumors with activating APC
mutations. Cancer Res.

[257]  Wharton KA (2003). Runnin' with the Dvl: proteins that associate with
Dsh/Dvl and their significance to Wnt signal transduction. Dev Biol.

[258]  Metcalfe C, Mendoza-Topaz C, Mieszczanek J, Bienz M (2010). Stability
elements in the LRP6 cytoplasmic tail confer efficient signalling
upon DIX-dependent polymerization. J Cell Sci.

[259]  Liu YT, Dan QJ, Wang J, Feng Y, Chen L, Liang J (2011). Molecular
basis of Wnt activation via the DIX domain protein Ccd1. J Biol Chem.

[260]  Schwarz-Romond T, Merrifield C, Nichols BJ, Bienz M (2005). The Wnt
signalling effector Dishevelled forms dynamic protein assemblies
rather than stable associations with cytoplasmic vesicles. J Cell Sci.

[261]  Smalley MJ, Signoret N, Robertson D, Tilley A, Hann A, Ewan K (2005). Dishevelled (Dvl-2) activates canonical Wnt signalling in the
absence of cytoplasmic puncta. J Cell Sci.

[262]  Simons M, Gault WJ, Gotthardt D, Rohatgi R, Klein TJ, Shao Y (2009). Electrochemical cues regulate assembly of the Frizzled/Dishevelled complex at the plasma membrane during planar
epithelial polarization. Nat Cell Biol.

[263]  Tauriello DV, Jordens I, Kirchner K, Slootstra JW, Kruitwagen T, Bouwman BA (2012). Wnt/beta-catenin signaling requires interaction
of the Dishevelled DEP domain and C terminus with a discontinuous
motif in Frizzled. Proc Natl Acad Sci U S A.

[264]  Punchihewa C, Ferreira AM, Cassell R, Rodrigues P, Fujii N (2009). Sequence
requirement and subtype specificity in the high-affinity interaction
between human frizzled and dishevelled proteins. Protein Sci.

[265]  Zhang L, Gao X, Wen J, Ning Y, Chen YG (2006). Dapper 1 antagonizes
Wnt signaling by promoting dishevelled degradation. J Biol Chem.

[266]  Chen H, Liu L, Ma B, Ma TM, Hou JJ, Xie GM (2011). Protein
kinase A-mediated 14-3-3 association impedes human Dapper1 to
promote dishevelled degradation. J Biol Chem.

[267]  Su Y, Zhang L, Gao X, Meng F, Wen J, Zhou H (2007). The evolutionally
conserved activity of Dapper2 in antagonizing TGF-beta
signaling. FASEB J.

[268]  Jiang X, Tan J, Li J, Kivimae S, Yang X, Zhuang L (2008). DACT3 is
an epigenetic regulator of Wnt/beta-catenin signaling in colorectal
cancer and is a therapeutic target of histone modifications. Cancer Cell.

[269]  Chen W, ten Berge D, Brown J, Ahn S, Hu LA, Miller WE (2003). Dishevelled 2 recruits beta-arrestin 2 to mediate Wnt5A-stimulated
endocytosis of Frizzled 4. Science.

[270]  Bryja V, Gradl D, Schambony A, Arenas E, Schulte G (2007). Beta-arrestin
is a necessary component of Wnt/beta-catenin signaling *in
vitro* and *in vivo*. Proc Natl Acad Sci U S A.

[271]  Yu A, Rual JF, Tamai K, Harada Y, Vidal M, He X (2007). Association
of Dishevelled with the clathrin AP-2 adaptor is required for
Frizzled endocytosis and planar cell polarity signaling. Dev Cell.

[272]  Klimowski LK, Garcia BA, Shabanowitz J, Hunt DF, Virshup DM (2006). Site-specific casein kinase 1epsilon-dependent phosphorylation of
Dishevelled modulates beta-catenin signaling. FEBS J.

[273]  Bryja V, Schulte G, Arenas E (2007). Wnt-3a utilizes a novel low dose and
rapid pathway that does not require casein kinase 1-mediated phosphorylation
of Dvl to activate beta-catenin. Cell Signal.

[274]  Bernatik O, Ganji RS, Dijksterhuis JP, Konik P, Cervenka I, Polonio 
T (2011). Sequential activation and inactivation of Dishevelled in the Wnt/beta-catenin pathway by casein kinases. J Biol Chem.

[275]  Itoh K, Brott BK, Bae GU, Ratcliffe MJ, Sokol SY (2005). Nuclear localization
is required for Dishevelled function in Wnt/beta-catenin signaling. J Biol.

[276]  Gao C, Cao W, Bao L, Zuo W, Xie G, Cai T (2010). Autophagy negatively
regulates Wnt signalling by promoting Dishevelled degradation. Nat Cell Biol.

[277]  Gan XQ, Wang JY, Xi Y, Wu ZL, Li YP, Li L (2008). Nuclear Dvl, c-Jun,
beta-catenin, and TCF form a complex leading to stabilization of
beta-catenin-TCF interaction. J Cell Biol.

[278]  Holloway KR, Calhoun TN, Saxena M, Metoyer CF, Kandler EF, Rivera CA (2010). SIRT1 regulates Dishevelled proteins and promotes
transient and constitutive Wnt signaling. Proc Natl Acad Sci U S A.

[279]  Lai SL, Chien AJ, Moon RT (2009). Wnt/Fz signaling and the cytoskeleton:
potential roles in tumorigenesis. Cell Res.

[280]  Krylova O, Messenger MJ, Salinas PC (2000). Dishevelled-1 regulates
microtubule stability: a new function mediated by glycogen synthase
kinase-3beta. J Cell Biol.

[281]  Ciani L, Krylova O, Smalley MJ, Dale TC, Salinas PC (2004). A divergent
canonical WNT-signaling pathway regulates microtubule dynamics:
dishevelled signals locally to stabilize microtubules. J Cell Biol.

[282]  Salinas PC (2007). Modulation of the microtubule cytoskeleton: a role for
a divergent canonical Wnt pathway. Trends Cell Biol.

[283]  Purro SA, Ciani L, Hoyos-Flight M, Stamatakou E, Siomou E, Salinas PC (2008). Wnt regulates axon behavior through changes in microtubule
growth directionality: a new role for adenomatous polyposis
coli. J Neurosci.

[284]  Lee HJ, Wang NX, Shi DL, Zheng JJ (2009). Sulindac inhibits canonical
Wnt signaling by blocking the PDZ domain of the protein Dishevelled. Angew Chem Int Ed Engl.

[285]  Shan J, Zheng JJ (2009). Optimizing Dvl PDZ domain inhibitor by exploring chemical space. J Comput Aided Mol Des.

[286]  Grandy D, Shan J, Zhang X, Rao S, Akunuru S, Li H (2009). Discovery
and characterization of a small molecule inhibitor of the PDZ
domain of dishevelled. J Biol Chem.

[287]  Zhang Y, Appleton BA, Wiesmann C, Lau T, Costa M, Hannoush RN (2009). Inhibition of Wnt signaling by Dishevelled PDZ peptides. Nat Chem Biol.

[288]  Shan J, Zhang X, Bao J, Cassell R, Zheng JJ (2012). Synthesis of potent
dishevelled PDZ domain inhibitors guided by virtual screening and
NMR studies. Chem Biol Drug Des.

[289]  Shan J, Shi DL, Wang J, Zheng J (2005). Identification of a specific inhibitor
of the dishevelled PDZ domain. Biochemistry.

[290]  Andersson ER (2012). The role of endocytosis in activating and regulating
signal transduction. Cell Mol Life Sci.

[291]  Li Y, Lu W, King TD, Liu CC, Bijur GN, Bu G (2010). Dkk1 stabilizes
Wnt co-receptor LRP6: implication for Wnt ligand-induced LRP6
down-regulation. PLoS One.

[292]  Gagliardi M, Piddini E, Vincent JP (2008). Endocytosis: a positive or a
negative influence on Wnt signalling?. Traffic.

[293]  Dubois L, Lecourtois M, Alexandre C, Hirst E, Vincent JP (2001). Regulated
endocytic routing modulates wingless signaling in Drosophila
embryos. Cell.

[294]  Zhang J, Barak LS, Winkler KE, Caron MG, Ferguson SS (1997). A central
role for beta-arrestins and clathrin-coated vesicle-mediated endocytosis
in beta2-adrenergic receptor resensitization. Differential
regulation of receptor resensitization in two distinct cell types. J Biol Chem.

[295]  Chen W, Hu LA, Semenov MV, Yanagawa S, Kikuchi A, Lefkowitz RJ (2001). beta-Arrestin1 modulates lymphoid enhancer factor
transcriptional activity through interaction with phosphorylated dishevelled
proteins. Proc Natl Acad Sci U S A.

[296]  Jiang Y, He X, Howe PH (2012). Disabled-2 (Dab2) inhibits Wnt/beta-catenin
signalling by binding LRP6 and promoting its internalization
through clathrin. EMBO J.

[297]  Yamamoto H, Sakane H, Yamamoto H, Michiue T, Kikuchi A (2008). Wnt3a and Dkk1 regulate distinct internalization pathways of LRP6
to tune the activation of beta-catenin signaling. Dev Cell.

[298]  Platta HW, Stenmark H (2011). Endocytosis and signaling. Curr Opin Cell Biol.

[299]  Fevrier B, Raposo G (2004). Exosomes: endosomal-derived vesicles shipping
extracellular messages. Curr Opin Cell Biol.

[300]  Simons M, Raposo G (2009). Exosomes--vesicular carriers for intercellular
communication. Curr Opin Cell Biol.

[301]  Schneider A, Simons M (2012). Exosomes: vesicular carriers for
intercellular communication in neurodegenerative disorders. Cell
Tissue Res.

[302]  Piddini E, Marshall F, Dubois L, Hirst E, Vincent JP (2005). Arrow
(LRP6) and Frizzled2 cooperate to degrade Wingless in Drosophila
imaginal discs. Development.

[303]  Hupalowska A, Miaczynska M (2012). The new faces of endocytosis in
signaling. Traffic.

[304]  Hay E, Nouraud A, Marie PJ (2009). N-cadherin negatively regulates osteoblast
proliferation and survival by antagonizing Wnt, ERK and
PI3K/Akt signalling. PLoS One.

[305]  Casagolda D, Del Valle-Perez B, Valls G, Lugilde E, Vinyoles M, Casado-Vela J (2010). A p120-catenin-CK1epsilon complex regulates
Wnt signaling. J Cell Sci.

[306]  Dupre-Crochet S, Figueroa A, Hogan C, Ferber EC, Bialucha CU, Adams J (2007). Casein kinase 1 is a novel negative regulator of E-cadherin-
based cell-cell contacts. Mol Cell Biol.

[307]  Greer YE, Rubin JS (2011). Casein kinase 1 delta functions at the centrosome
to mediate Wnt-3a-dependent neurite outgrowth. J Cell Biol.

[308]  Cheong JK, Nguyen TH, Wang H, Tan P, Voorhoeve PM, Lee SH (2011). IC261 induces cell cycle arrest and apoptosis of human cancer
cells via CK1delta/epsilon and Wnt/beta-catenin independent inhibition
of mitotic spindle formation. Oncogene.

[309]  Park JI, Ji H, Jun S, Gu D, Hikasa H, Li L (2006). Frodo links Dishevelled
to the p120-catenin/Kaiso pathway: distinct catenin subfamilies
promote Wnt signals. Dev Cell.

[310]  Cox RT, Kirkpatrick C, Peifer M (1996). Armadillo is required for adherens
junction assembly, cell polarity, and morphogenesis during
Drosophila embryogenesis. J Cell Biol.

[311]  Brembeck FH, Rosario M, Birchmeier W (2006). Balancing cell adhesion
and Wnt signaling, the key role of beta-catenin. Curr Opin Genet
Dev.

[312]  Thiery JP (2003). Cell adhesion in development: a complex signaling network. Curr Opin Genet Dev.

[313]  Stockinger A, Eger A, Wolf J, Beug H, Foisner R (2001). E-cadherin regulates
cell growth by modulating proliferation-dependent beta-catenin
transcriptional activity. J Cell Biol.

[314]  Gottardi CJ, Wong E, Gumbiner BM (2001). E-cadherin suppresses cellular
transformation by inhibiting beta-catenin signaling in an adhesion-
independent manner. J Cell Biol.

[315]  Orsulic S, Huber O, Aberle H, Arnold S, Kemler R (1999). E-cadherin
binding prevents beta-catenin nuclear localization and beta-catenin/
LEF-1-mediated transactivation. J Cell Sci.

[316]  Brooke MA, Nitoiu D, Kelsell DP (2012). Cell-cell connectivity: desmosomes and disease. J Pathol.

[317]  Gumbiner BM (1996). Cell adhesion: the molecular basis of tissue architecture
and morphogenesis. Cell.

[318]  Yeaman C, Grindstaff KK, Nelson WJ (1999). New perspectives on
mechanisms involved in generating epithelial cell polarity. Physiol Rev.

[319]  Dejana E (2004). Endothelial cell-cell junctions: happy together. Nat Rev Mol Cell Biol.

[320]  Gravdal K, Halvorsen OJ, Haukaas SA, Akslen LA (2007). A switch from
E-cadherin to N-cadherin expression indicates epithelial to mesenchymal
transition and is of strong and independent importance for
the progress of prostate cancer. Clin Cancer Res.

[321]  Wheelock MJ, Shintani Y, Maeda M, Fukumoto Y, Johnson KR (2008). Cadherin switching. J Cell Sci.

[322]  Hazan RB, Kang L, Whooley BP, Borgen PI (1997). N-cadherin promotes
adhesion between invasive breast cancer cells and the stroma. Cell Adhes Commun.

[323]  Hazan RB, Kang L, Roe S, Borgen PI, Rimm DL (1997). Vinculin is associated
with the E-cadherin adhesion complex. J Biol Chem.

[324]  Knudsen KA, Sauer C, Johnson KR, Wheelock MJ (2005). Effect of N-cadherin
misexpression by the mammary epithelium in mice. J Cell Biochem.

[325]  Hulit J, Suyama K, Chung S, Keren R, Agiostratidou G, Shan W (2007). N-cadherin signaling potentiates mammary tumor metastasis via
enhanced extracellular signal-regulated kinase activation. Cancer Res.

[326]  Behrens J, Weidner KM, Frixen UH, Schipper JH, Sachs M, Arakaki N (1991). The role of E-cadherin and scatter factor in tumor invasion
and cell motility. EXS.

[327]  Frixen UH, Behrens J, Sachs M, Eberle G, Voss B, Warda A (1991). E-cadherin-mediated cell-cell adhesion prevents invasiveness of
human carcinoma cells. J Cell Biol.

[328]  Nagar B, Overduin M, Ikura M, Rini JM (1996). Structural basis of calcium-
induced E-cadherin rigidification and dimerization. Nature.

[329]  Simcha I, Geiger B, Yehuda-Levenberg S, Salomon D, Ben-Ze'ev A (1996). Suppression of tumorigenicity by plakoglobin: an augmenting
effect of N-cadherin. J Cell Biol.

[330]  Ishiyama N, Lee SH, Liu S, Li GY, Smith MJ, Reichardt LF (2010). Dynamic and static interactions between p120 catenin and E-cadherin
regulate the stability of cell-cell adhesion. Cell.

[331]  Davis MA, Ireton RC, Reynolds AB (2003). A core function for p120-catenin in cadherin turnover. J Cell Biol.

[332]  Xiao K, Allison DF, Buckley KM, Kottke MD, Vincent PA, Faundez V (2003). Cellular levels of p120 catenin function as a set point
for cadherin expression levels in microvascular endothelial cells. J Cell Biol.

[333]  Kowalczyk AP, Reynolds AB (2004). Protecting your tail: regulation of
cadherin degradation by p120-catenin. Curr Opin Cell Biol.

[334]  Ireton RC, Davis MA, van Hengel J, Mariner DJ, Barnes K, Thoreson MA (2002). A novel role for p120 catenin in E-cadherin
function. J Cell Biol.

[335]  Reynolds AB (2007). p120-catenin: Past and present. Biochim Biophys Acta.

[336]  Nagafuchi A, Takeichi M, Tsukita S (1991). The 102 kd cadherin-associated
protein: similarity to vinculin and posttranscriptional
regulation of expression. Cell.

[337]  Takeichi M (1991). Cadherin cell adhesion receptors as a morphogenetic regulator. Science.

[338]  Gumbiner BM, McCrea PD (1993). Catenins as mediators of the cytoplasmic
functions of cadherins. J Cell Sci Suppl.

[339]  Yamada S, Pokutta S, Drees F, Weis WI, Nelson WJ (2005). Deconstructing
the cadherin-catenin-actin complex. Cell.

[340]  Choi HJ, Gross JC, Pokutta S, Weis WI (2009). Interactions of plakoglobin
and beta-catenin with desmosomal cadherins: basis of selective exclusion
of alpha- and beta-catenin from desmosomes. J Biol Chem.

[341]  Huber AH, Weis WI (2001). The structure of the beta-catenin/E-cadherin
complex and the molecular basis of diverse ligand recognition by
beta-catenin. Cell.

[342]  Pokutta S, Weis WI (2000). Structure of the dimerization and beta-catenin-binding
region of alpha-catenin. Mol Cell.

[343]  Piedra J, Miravet S, Castano J, Palmer HG, Heisterkamp N, Garcia de HA (2003). p120 Catenin-associated Fer and Fyn tyrosine kinases
regulate beta-catenin Tyr-142 phosphorylation and beta-catenin-alpha-catenin Interaction. Mol Cell Biol.

[344]  Kajiguchi T, Katsumi A, Tanizaki R, Kiyoi H, Naoe T (2012). Y654 of
beta-catenin is essential for FLT3/ITD-related tyrosine phosphorylation
and nuclear localization of beta-catenin. Eur J Haematol.

[345]  Piedra J, Martinez D, Castano J, Miravet S, Dunach M, de Herreros 
AG (2001). Regulation of beta-catenin structure and activity by tyrosine
phosphorylation. J Biol Chem.

[346]  van Veelen W, Le NH, Helvensteijn W, Blonden L, Theeuwes M, Bakker ER (2011). beta-catenin tyrosine 654 phosphorylation increases
Wnt signalling and intestinal tumorigenesis. Gut.

[347]  Hino S, Tanji C, Nakayama KI, Kikuchi A (2005). Phosphorylation of
beta-catenin by cyclic AMP-dependent protein kinase stabilizes
beta-catenin through inhibition of its ubiquitination. Mol Cell Biol.

[348]  Taurin S, Sandbo N, Qin Y, Browning D, Dulin NO (2006). Phosphorylation
of beta-catenin by cyclic AMP-dependent protein kinase. J Biol Chem.

[349]  Tian Q, Feetham MC, Tao WA, He XC, Li L, Aebersold R (2004). Proteomic analysis identifies that 14-3-3zeta interacts with beta-catenin
and facilitates its activation by Akt. Proc Natl Acad Sci U S A.

[350]  Agarwal A, Das K, Lerner N, Sathe S, Cicek M, Casey G (2005). The
AKT/I kappa B kinase pathway promotes angiogenic/metastatic
gene expression in colorectal cancer by activating nuclear factor-kappa
B and beta-catenin. Oncogene.

[351]  Fang D, Hawke D, Zheng Y, Xia Y, Meisenhelder J, Nika H (2007). Phosphorylation of beta-catenin by AKT promotes beta-catenin
transcriptional activity. J Biol Chem.

[352]  Roura S, Miravet S, Piedra J, Garcia de Herreros A, Dunach M (1999). Regulation of E-cadherin/Catenin association by tyrosine phosphorylation. J Biol Chem.

[353]  Nam JS, Ino Y, Sakamoto M, Hirohashi S (2002). Src family kinase inhibitor
PP2 restores the E-cadherin/catenin cell adhesion system in human
cancer cells and reduces cancer metastasis. Clin Cancer Res.

[354]  Genda T, Sakamoto M, Ichida T, Asakura H, Hirohashi S (2000). Loss of
cell-cell contact is induced by integrin-mediated cell-substratum
adhesion in highly-motile and highly-metastatic hepatocellular carcinoma
cells. Lab Invest.

[355]  Menke A, Philippi C, Vogelmann R, Seidel B, Lutz MP, Adler G (2001). Down-regulation of E-cadherin gene expression by collagen
type I and type III in pancreatic cancer cell lines. Cancer Res.

[356]  Coluccia AM, Benati D, Dekhil H, De Filippo A, Lan C, Gambacorti-Passerini C (2006). SKI-606 decreases growth and motility of colorectal
cancer cells by preventing pp60(c-Src)-dependent tyrosine
phosphorylation of beta-catenin and its nuclear signaling. Cancer Res.

[357]  Vultur A, Buettner R, Kowolik C, Liang W, Smith D, Boschelli F (2008). SKI-606 (bosutinib), a novel Src kinase inhibitor, suppresses
migration and invasion of human breast cancer cells. Mol Cancer
Ther.

[358]  Daud AI, Krishnamurthi SS, Saleh MN, Gitlitz BJ, Borad MJ, Gold 
PJ (2012). Phase I study of bosutinib, a src/abl tyrosine kinase inhibitor,
administered to patients with advanced solid tumors. Clin Cancer Res.

[359]  Marambaud P, Shioi J, Serban G, Georgakopoulos A, Sarner S, 
Nagy V (2002). A presenilin-1/gamma-secretase cleavage releases the
E-cadherin intracellular domain and regulates disassembly of adherens
junctions. EMBO J.

[360]  Serban G, Kouchi Z, Baki L, Georgakopoulos A, Litterst CM, Shioi 
J (2005). Cadherins mediate both the association between PS1 and
beta-catenin and the effects of PS1 on beta-catenin stability. J Biol
Chem.

[361] Sanson B, White P, Vincent JP (1996). Uncoupling cadherin-based adhesion
from wingless signalling in Drosophila. Nature.

[362]  Sadot E, Simcha I, Shtutman M, Ben-Ze'ev A, Geiger B (1998). Inhibition
of beta-catenin-mediated transactivation by cadherin derivatives. Proc Natl Acad Sci U S A.

[363]  Salahshor S, Naidoo R, Serra S, Shih W, Tsao MS, Chetty R (2008). Frequent accumulation of nuclear E-cadherin and alterations in the
Wnt signaling pathway in esophageal squamous cell carcinomas. Mod Pathol.

[364]  Elston MS, Gill AJ, Conaglen JV, Clarkson A, Cook RJ, Little NS (2009). Nuclear accumulation of e-cadherin correlates with loss of cytoplasmic
membrane staining and invasion in pituitary adenomas. J
Clin Endocrinol Metab.

[365]  Rios-Doria J, Day KC, Kuefer R, Rashid MG, Chinnaiyan AM, 
Rubin MA (2003). The role of calpain in the proteolytic cleavage of
E-cadherin in prostate and mammary epithelial cells. J Biol Chem.

[366]  Pan FY, Zhang SZ, Xu N, Meng FL, Zhang HX, Xue B (2010). Beta-catenin
signaling involves HGF-enhanced HepG2 scattering
through activating MMP-7 transcription. Histochem Cell Biol.

[367]  Aberle H, Schwartz H, Kemler R (1996). Cadherin-catenin complex: protein
interactions and their implications for cadherin function. J Cell
Biochem.

[368]  Aberle H, Schwartz H, Hoschuetzky H, Kemler R (1996). Single amino
acid substitutions in proteins of the armadillo gene family abolish
their binding to alpha-catenin. J Biol Chem.

[369]  Choi KH, Park MW, Lee SY, Jeon MY, Kim MY, Lee HK (2006). Intracellular expression of the T-cell factor-1 RNA aptamer as an
intramer. Mol Cancer Ther.

[370]  Hoschuetzky H, Aberle H, Kemler R (1994). Beta-catenin mediates the
interaction of the cadherin-catenin complex with epidermal growth
factor receptor. J Cell Biol.

[371]  Maetzel D, Denzel S, Mack B, Canis M, Went P, Benk M (2009). Nuclear signalling by tumour-associated antigen EpCAM. Nat Cell
Biol.

[372]  Denzel S, Maetzel D, Mack B, Eggert C, Barr G, Gires O (2009). Initial
activation of EpCAM cleavage via cell-to-cell contact. BMC Cancer.

[373]  Blaschuk OW, Devemy E (2009). Cadherins as novel targets for anti-cancer
therapy. Eur J Pharmacol.

[374]  Yarom N, Stewart D, Malik R, Wells J, Avruch L, Jonker DJ (2012). Phase
I Clinical Trial of Exherin (ADH-1) in Patients with Advanced
Solid Tumors. Curr Clin Pharmacol.

[375] Iozzo MV (2000). Proteoglycans: structure, biology and molecular interactions.
Gallagher JTLM, editor. Molecular structure of Heparan Sulfate
and interactions with growth factors and morphogens. New York, Marcel Dekker Inc. Ref Type: Generic.

[376]  Zhang YW, Vande Woude GF (2003). HGF/SF-met signaling in the control
of branching morphogenesis and invasion. J Cell Biochem.

[377]  Gao CF, Vande Woude GF (2005). HGF/SF-Met signaling in tumor progression. Cell Res.

[378]  Gherardi E, Birchmeier W, Birchmeier C, Vande WG (2012). Targeting
MET in cancer: rationale and progress. Nat Rev Cancer.

[379]  Vermeulen L, de Sousa E, Melo van der Heijden M, Cameron K, de Jong JH, Borovski T (2010). Wnt activity defines colon cancer
stem cells and is regulated by the microenvironment. Nat Cell Biol.

[380]  Monga SP, Mars WM, Pediaditakis P, Bell A, Mule K, Bowen WC (2002). Hepatocyte growth factor induces Wnt-independent nuclear
translocation of beta-catenin after Met-beta-catenin dissociation in
hepatocytes. Cancer Res.

[381]  Nelson WJ, Nusse R (2004). Convergence of Wnt, beta-catenin, and cadherin
pathways. Science.

[382]  Previdi S, Maroni P, Matteucci E, Broggini M, Bendinelli P, Desiderio MA (2010). Interaction between human-breast cancer metastasis and
bone microenvironment through activated hepatocyte growth factor/
Met and beta-catenin/Wnt pathways. Eur J Cancer.

[383]  Sharma M, Jamieson C, Johnson M, Molloy MP, Henderson BR (2012). Specific armadillo repeat sequences facilitate beta-catenin nuclear
transport in live cells via direct binding to nucleoporins Nup62,
Nup153, and RanBP2/Nup358. J Biol Chem.

[384]  Kajiguchi T, Chung EJ, Lee S, Stine A, Kiyoi H, Naoe T (2007). FLT3 regulates beta-catenin tyrosine phosphorylation, nuclear localization,
and transcriptional activity in acute myeloid leukemia
cells. Leukemia.

[385]  Zhou L, An N, Haydon RC, Zhou Q, Cheng H, Peng Y (2003). Tyrosine
kinase inhibitor STI-571/Gleevec down-regulates the beta-catenin
signaling activity. Cancer Lett.

[386]  Christensen JG, Schreck R, Burrows J, Kuruganti P, Chan E, Le P (2003). A selective small molecule inhibitor of c-Met kinase inhibits
c-Met-dependent phenotypes *in vitro* and exhibits cytoreductive antitumor
activity *in vivo*. Cancer Res.

[387]  Cianfrocca R, Tocci P, Spinella F, Di C V, Bagnato A, Rosano L (2012). The endothelin A receptor and epidermal growth factor receptor
signaling converge on beta-catenin to promote ovarian cancer metastasis. Life Sci.

[388]  Danilkovitch-Miagkova A (2003). Oncogenic signaling pathways activated
by RON receptor tyrosine kinase. Curr Cancer Drug Targets.

[389]  Grotegut S, Von Schweinitz D, Christofori G, Lehembre F (2006). Hepatocyte
growth factor induces cell scattering through MAPK/Egr-1-mediated upregulation of Snail. EMBO J.

[390]  Leroy P, Mostov KE (2007). Slug is required for cell survival during partial
epithelial-mesenchymal transition of HGF-induced tubulogenesis. Mol Biol Cell.

[391]  Ranganathan S, Tan X, Monga SP (2005). beta-Catenin and met deregulation
in childhood Hepatoblastomas. Pediatr Dev Pathol.

[392]  Lickert H, Bauer A, Kemler R, Stappert J (2000). Casein kinase II phosphorylation
of E-cadherin increases E-cadherin/beta-catenin interaction
and strengthens cell-cell adhesion. J Biol Chem.

[393]  Du C, Jaggi M, Zhang C, Balaji KC (2009). Protein kinase D1-mediated
phosphorylation and subcellular localization of beta-catenin. Cancer Res.

[394]  Li J, Sutter C, Parker DS, Blauwkamp T, Fang M, Cadigan KM (2007). CBP/p300 are bimodal regulators of Wnt signaling. EMBO J.

[395]  Huber O, Korn R, McLaughlin J, Ohsugi M, Herrmann BG, Kemler R (1996). Nuclear localization of beta-catenin by interaction with transcription
factor LEF-1. Mech Dev.

[396]  Arce L, Yokoyama NN, Waterman ML (2006). Diversity of LEF/TCF
action in development and disease. Oncogene.

[397]  Hoppler S, Kavanagh CL (2007). Wnt signalling: variety at the core. J Cell Sci.

[398]  Solberg N, Machon O, Machonova O, Krauss S (2012). Mouse Tcf3 represses
canonical Wnt signaling by either competing for beta-catenin
binding or through occupation of DNA-binding sites. Mol
Cell Biochem.

[399]  Van de Wetering M, Castrop J, Korinek V, Clevers H (1996). Extensive
alternative splicing and dual promoter usage generate Tcf-1 protein
isoforms with differential transcription control properties. Mol Cell
Biol.

[400]  Brantjes H, Roose J, van de Wetering M, Clevers H (2001). All Tcf HMG
box transcription factors interact with Groucho-related co-repressors. Nucleic Acids Res.

[401]  Archbold HC, Yang YX, Chen L, Cadigan KM (2012). How do they do
Wnt they do?: regulation of transcription by the Wnt/beta-catenin
pathway. Acta Physiol (Oxf).

[402]  Cadigan KM (2012). TCFs and Wnt/beta-catenin signaling: more than one way to throw the switch. Curr Top Dev Biol.

[403]  Daniels DL, Weis WI (2005). Beta-catenin directly displaces Groucho/TLE repressors from Tcf/Lef in Wnt-mediated transcription activation. Nat Struct Mol Biol.

[404]  Poy F, Lepourcelet M, Shivdasani RA, Eck MJ (2001). Structure of a human Tcf4-beta-catenin complex. Nat Struct Biol.

[405]  Zhurinsky J, Shtutman M, Ben-Ze'ev A (2000). Differential mechanisms of LEF/TCF family-dependent transcriptional activation by beta-catenin and plakoglobin. Mol Cell Biol.

[406]  Miravet S, Piedra J, Miro F, Itarte E, Garcia de Herreros A, Dunach M (2002). The transcriptional factor Tcf-4 contains different binding sites for beta-catenin and plakoglobin. J Biol Chem.

[407]  Maeda O, Usami N, Kondo M, Takahashi M, Goto H, Shimokata K (2004). Plakoglobin (gamma-catenin) has TCF/LEF family-dependent transcriptional activity in beta-catenin-deficient cell line. Oncogene.

[408]  Kolligs FT, Kolligs B, Hajra KM, Hu G, Tani M, Cho KR (2000). gamma-catenin is regulated by the APC tumor suppressor and its oncogenic activity is distinct from that of beta-catenin. Genes Dev.

[409]  Karnovsky A, Klymkowsky MW (1995). Anterior axis duplication in Xenopus induced by the over-expression of the cadherin-binding protein plakoglobin. Proc Natl Acad Sci U S A.

[410]  Daniel JM, Spring CM, Crawford HC, Reynolds AB, Baig A (2002). The p120(ctn)-binding partner Kaiso is a bi-modal DNA-binding protein that recognizes both a sequence-specific consensus and methylated CpG dinucleotides. Nucleic Acids Res.

[411]  Wang HY, Wang YJ, Cui MJ, Gu CM, Yang LZ, Zhao Y (2011). Hepatocyte growth factor-induced amelioration in renal interstitial fibrosis is associated with reduced expression of alpha-smooth muscle actin and transforming growth factor-beta1. Indian J Biochem Biophys.

[412]  Park JI, Kim SW, Lyons JP, Ji H, Nguyen TT, Cho K (2005). Kaiso/p120-catenin and TCF/beta-catenin complexes coordinately regulate canonical Wnt gene targets. Dev Cell.

[413]  Spring CM, Kelly KF, O'Kelly I, Graham M, Crawford HC, Daniel JM (2005). The catenin p120ctn inhibits Kaiso-mediated transcriptional repression of the beta-catenin/TCF target gene matrilysin. Exp Cell Res.

[414]  Bauer A, Huber O, Kemler R (1998). Pontin52, an interaction partner of beta-catenin, binds to the TATA box binding protein. Proc Natl Acad Sci U S A.

[415]  Hecht A, Litterst CM, Huber O, Kemler R (1999). Functional characterization of multiple transactivating elements in beta-catenin, some of which interact with the TATA-binding protein *in vitro*. J Biol Chem.

[416]  Kramps T, Peter O, Brunner E, Nellen D, Froesch B, Chatterjee S (2002). Wnt/wingless signaling requires BCL9/legless-mediated recruitment of pygopus to the nuclear beta-catenin-TCF complex. Cell.

[417]  Townsley FM, Cliffe A, Bienz M (2004). Pygopus and Legless target Armadillo/beta-catenin to the nucleus to enable its transcriptional co-activator function. Nat Cell Biol.

[418]  Takemaru K, Yamaguchi S, Lee YS, Zhang Y, Carthew RW, Moon RT (2003). Chibby, a nuclear beta-catenin-associated antagonist of the Wnt/Wingless pathway. Nature.

[419]  Li FQ, Mofunanya A, Harris K, Takemaru K (2008). Chibby cooperates with 14-3-3 to regulate beta-catenin subcellular distribution and signaling activity. J Cell Biol.

[420]  Chua EL, Young L, Wu WM, Turtle JR, Dong Q (2000). Cloning of TC-1 (C8orf4), a novel gene found to be overexpressed in thyroid cancer. Genomics.

[421]  Sunde M, McGrath KC, Young L, Matthews JM, Chua EL, Mackay JP (2004). TC-1 is a novel tumorigenic and natively disordered protein associated with thyroid cancer. Cancer Res.

[422]  Zhang C, Cho K, Huang Y, Lyons JP, Zhou X, Sinha K (2008). Inhibition of Wnt signaling by the osteoblast-specific transcription factor Osterix. Proc Natl Acad Sci U S A.

[423]  Torres MA, Nelson WJ (2000). Colocalization and redistribution of dishevelled and actin during Wnt-induced mesenchymal morphogenesis. J Cell Biol.

[424]  Hammerlein A, Weiske J, Huber O (2005). A second protein kinase CK1-mediated step negatively regulates Wnt signalling by disrupting the lymphocyte enhancer factor-1/beta-catenin complex. Cell Mol Life Sci.

[425]  Wang S, Jones KA (2006). CK2 controls the recruitment of Wnt regulators to target genes *in vivo*. Curr Biol.

[426]  Jin Y, Lu Z, Ding K, Li J, Du X, Chen C (2010). Antineoplastic mechanisms of niclosamide in acute myelogenous leukemia stem cells: inactivation of the NF-kappaB pathway and generation of reactive oxygen species. Cancer Res.

[427]  Sun J, Weis WI (2011). Biochemical and structural characterization of beta-catenin interactions with nonphosphorylated and CK2-phosphorylated Lef-1. J Mol Biol.

[428]  Ishitani T, Ninomiya-Tsuji J, Nagai S, Nishita M, Meneghini M, Barker N (1999). The TAK1-NLK-MAPK-related pathway antagonizes signalling between beta-catenin and transcription factor TCF. Nature.

[429]  Ishitani T (2012). Context-dependent dual and opposite roles of nemo-like kinase in the Wnt/beta-catenin signaling. Cell Cycle.

[430]  Zeng YA, Verheyen EM (2004). Nemo is an inducible antagonist of Wingless signaling during Drosophila wing development. Development.

[431]  Ishitani T, Ninomiya-Tsuji J, Matsumoto K (2003). Regulation of lymphoid enhancer factor 1/T-cell factor by mitogen-activated protein kinase-related Nemo-like kinase-dependent phosphorylation in Wnt/beta-catenin signaling. Mol Cell Biol.

[432]  Ota S, Ishitani S, Shimizu N, Matsumoto K, Itoh M, Ishitani T (2012). NLK positively regulates Wnt/beta-catenin signalling by phosphorylating LEF1 in neural progenitor cells. EMBO J.

[433]  Sachdev S, Bruhn L, Sieber H, Pichler A, Melchior F, Grosschedl R (2001). PIASy, a nuclear matrix-associated SUMO E3 ligase, represses LEF1 activity by sequestration into nuclear bodies. Genes Dev.

[434]  Emami KH, Nguyen C, Ma H, Kim DH, Jeong KW, Eguchi M (2004). A small molecule inhibitor of beta-catenin/CREB-binding protein transcription [corrected]. Proc Natl Acad Sci U S A.

[435]  Zhou B, Liu Y, Kahn M, Ann DK, Han A, Wang H (2012). Interactions between beta-catenin and transforming growth factor-beta signaling pathways mediate epithelial-mesenchymal transition and are dependent on the transcriptional co-activator cAMP-response element-binding protein (CREB)-binding protein (CBP). J Biol Chem.

[436]  Takahashi-Yanaga F, Kahn M (2010). Targeting Wnt signaling: can we safely eradicate cancer stem cells?. Clin Cancer Res.

[437]  Lu D, Liu JX, Endo T, Zhou H, Yao S, Willert K (2009). Ethacrynic acid exhibits selective toxicity to chronic lymphocytic leukemia cells by inhibition of the Wnt/beta-catenin pathway. PLoS One.

[438]  Schmidt M, Kim Y, Gast SM, Endo T, Lu D, Carson D (2011). Increased *in vivo* efficacy of lenalidomide and thalidomide by addition of ethacrynic acid. In Vivo.

[439]  Jin G, Lu D, Yao S, Wu CC, Liu JX, Carson DA (2009). Amide derivatives of ethacrynic acid: synthesis and evaluation as antagonists of Wnt/beta-catenin signaling and CLL cell survival. Bioorg Med Chem Lett.

[440]  Lepourcelet M, Chen YN, France DS, Wang H, Crews P, Petersen F (2004). Small-molecule antagonists of the oncogenic Tcf/beta-catenin protein complex. Cancer Cell.

[441]  Barker N, Clevers H (2006). Mining the Wnt pathway for cancer therapeutics. Nat Rev Drug Discov.

[442]  Wei W, Chua MS, Grepper S, So S (2010). Small molecule antagonists of Tcf4/beta-catenin complex inhibit the growth of HCC cells *in vitro* and *in vivo*. Int J Cancer.

[443]  Trosset JY, Dalvit C, Knapp S, Fasolini M, Veronesi M, Mantegani S (2006). Inhibition of protein-protein interactions: the discovery of druglike beta-catenin inhibitors by combining virtual and biophysical screening. Proteins.

[444]  Tian W, Han X, Yan M, Xu Y, Duggineni S, Lin N (2012). Structure-based discovery of a novel inhibitor targeting the beta-catenin/Tcf4 interaction. Biochemistry.

[445]  Maxwell PH, Wiesener MS, Chang GW, Clifford SC, Vaux EC, Cockman ME (1999). The tumour suppressor protein VHL targets hypoxia-inducible factors for oxygen-dependent proteolysis. Nature.

[446]  Rubin JS, Bottaro DP (2007). Loss of secreted frizzled-related protein-1 expression in renal cell carcinoma reveals a critical tumor suppressor function. Clin Cancer Res.

[447]  Burrows N, Babur M, Resch J, Williams KJ, Brabant G (2011). Hypoxia-inducible factor in thyroid carcinoma. J Thyroid Res.

[448]  Semenza GL (2003). Targeting HIF-1 for cancer therapy. Nat Rev Cancer.

[449]  Burrows N, Babur M, Resch J, Ridsdale S, Mejin M, Rowling EJ (2011). GDC-0941 inhibits metastatic characteristics of thyroid carcinomas by targeting both the phosphoinositide-3 kinase (PI3K) and hypoxia-inducible factor-1alpha (HIF-1alpha) pathways. J Clin Endocrinol Metab.

[450]  Kaidi A, Williams AC, Paraskeva C (2007). Interaction between beta-catenin and HIF-1 promotes cellular adaptation to hypoxia. Nat Cell Biol.

[451]  Mazumdar J, O'Brien WT, Johnson RS, LaManna JC, Chavez JC, Klein PS (2010). O2 regulates stem cells through Wnt/beta-catenin signalling. Nat Cell Biol.

[452]  Newton IP, Kenneth NS, Appleton PL, Nathke I, Rocha S (2010). Adenomatous polyposis coli and hypoxia-inducible factor-1{alpha} have an antagonistic connection. Mol Biol Cell.

[453]  Kawabata A (2011). Prostaglandin E2 and pain--an update. Biol Pharm Bull.

[454]  Brabletz T, Hlubek F, Spaderna S, Schmalhofer O, Hiendlmeyer E, Jung A (2005). Invasion and metastasis in colorectal cancer: epithelial-mesenchymal transition, mesenchymal-epithelial transition, stem cells and beta-catenin. Cells Tissues Organs.

[455]  Buchanan FG, DuBois RN (2006). Connecting COX-2 and Wnt in cancer. Cancer Cell.

[456]  Fang X, Yu SX, Lu Y, Bast RC, Woodgett JR, Mills GB (2000). Phosphorylation and inactivation of glycogen synthase kinase 3 by protein kinase A. Proc Natl Acad Sci U S A.

[457]  Smartt HJ, Greenhough A, Ordonez-Moran P, Talero E, Cherry CA, Wallam CA (2011). beta-catenin represses expression of the tumour suppressor 15-prostaglandin dehydrogenase in the normal intestinal epithelium and colorectal tumour cells. Gut.

[458]  His LC, Angerman-Stewart J, Eling TE (1999). Introduction of full-length APC modulates cyclooxygenase-2 expression in HT-29 human colorectal carcinoma cells at the translational level. Carcinogenesis.

[459]  George SJ (2008). Wnt pathway: a new role in regulation of inflammation. Arterioscler Thromb Vasc Biol.

[460]  Din FV, Theodoratou E, Farrington SM, Tenesa A, Barnetson RA, Cetnarskyj R (2010). Effect of aspirin and NSAIDs on risk and survival from colorectal cancer. Gut.

[461]  Brudvik KW, Paulsen JE, Aandahl EM, Roald B, Tasken K (2011). Protein kinase A antagonist inhibits beta-catenin nuclear translocation, c-Myc and COX-2 expression and tumor promotion in Apc(Min/+) mice. Mol Cancer.

[462]  Massague J (2000). How cells read TGF-beta signals. Nat Rev Mol Cell Biol.

[463]  Shi Y, Massague J (2003). Mechanisms of TGF-beta signaling from cell membrane to the nucleus. Cell.

[464]  Liu W, Rui H, Wang J, Lin S, He Y, Chen M (2006). Axin is a scaffold protein in TGF-beta signaling that promotes degradation of Smad7 by Arkadia. EMBO J.

[465]  Imamura T, Takase M, Nishihara A, Oeda E, Hanai J, Kawabata M (1997). Smad6 inhibits signalling by the TGF-beta superfamily. Nature.

[466]  Kavsak P, Rasmussen RK, Causing CG, Bonni S, Zhu H, Thomsen GH (2000). Smad7 binds to Smurf2 to form an E3 ubiquitin ligase that targets the TGF beta receptor for degradation. Mol Cell.

[467]  Kim S, Jho EH (2010). The protein stability of Axin, a negative regulator of Wnt signaling, is regulated by Smad ubiquitination regulatory factor 2 (Smurf2). J Biol Chem.

[468]  Han G, Li AG, Liang YY, Owens P, He W, Lu S (2006). Smad7-induced beta-catenin degradation alters epidermal appendage development. Dev Cell.

[469]  Edlund S, Lee SY, Grimsby S, Zhang S, Aspenstrom P, Heldin CH (2005). Interaction between Smad7 and beta-catenin: importance for transforming growth factor beta-induced apoptosis. Mol Cell Biol.

[470]  Tang Y, Liu Z, Zhao L, Clemens TL, Cao X (2008). Smad7 stabilizes beta-catenin binding to E-cadherin complex and promotes cell-cell adhesion. J Biol Chem.

[471]  DiVito KA, Trabosh VA, Chen YS, Chen Y, Albanese C, Javelaud D (2010). Smad7 restricts melanoma invasion by restoring N-cadherin expression and establishing heterotypic cell-cell interactions *in vivo*. Pigment Cell Melanoma Res.

[472]  Yang L, Lin C, Liu ZR (2006). P68 RNA helicase mediates PDGF-induced epithelial mesenchymal transition by displacing Axin from beta-catenin. Cell.

[473]  Iqbal S, Zhang S, Driss A, Liu ZR, Kim HR, Wang Y (2012). PDGF upregulates Mcl-1 through activation of β-catenin and HIF-1α-dependent signaling in human prostate cancer cells. PLoS One.

[474]  Dufourcq P, Descamps B, Tojais NF, Leroux L, Oses P, Daret D (2008). Secreted frizzled-related protein-1 enhances mesenchymal stem cell function in angiogenesis and contributes to neovessel maturation. Stem Cells.

[475]  Kwon C, Cheng P, King IN, Andersen P, Shenje L, Nigam V (2011). Notch post-translationally regulates beta-catenin protein in stem and progenitor cells. Nat Cell Biol.

[476]  Katoh M (2007). Networking of WNT, FGF, Notch, BMP, and Hedgehog signaling pathways during carcinogenesis. Stem Cell Rev.

[477]  Casas-Selves M, Kim J, Zhang Z, Helfrich BA, Gao D, Porter CC (2012). Tankyrase and the canonical Wnt pathway protect lung cancer cells from EGFR inhibition. Cancer Res.

[478]  Tenbaum SP, Ordonez-Moran P, Puig I, Chicote I, Arques O, Landolfi S (2012). Beta-catenin confers resistance to PI3K and AKT inhibitors and subverts FOXO3a to promote metastasis in colon cancer. Nat Med.

[479]  Boon EM, Keller JJ, Wormhoudt TA, Giardiello FM, Offerhaus GJ, van der Neut R (2004). Sulindac targets nuclear beta-catenin accumulation and Wnt signalling in adenomas of patients with familial adenomatous polyposis and in human colorectal cancer cell lines. Br J Cancer.

